# Malaria is the leading cause of acute kidney injury among a Zambian paediatric renal service cohort retrospectively evaluated for aetiologies, predictors of the need for dialysis, and outcomes

**DOI:** 10.1371/journal.pone.0293037

**Published:** 2023-10-25

**Authors:** Chisambo Mwaba, Sody Munsaka, Bruce Bvulani, David Mwakazanga, Brian Chanda Chiluba, Kaiser Fitzwanga, Evans Mpabalwani

**Affiliations:** 1 Department of Paediatrics and Child Health, School of Medicine, University of Zambia, Lusaka, Zambia; 2 Department of Paediatrics, University Teaching Hospitals-Children’s Hospital, Lusaka, Zambia; 3 Department of Biomedical Sciences, School of Health Sciences, University of Zambia, Lusaka, Zambia; 4 Department of Paediatric Surgery, University Teaching Hospitals-Adult Hospital, Lusaka, Zambia; 5 Department of Public Health, Tropical Diseases Research Centre, 6-7^th^ Floors Ndola Teaching Hospital, Ndola, Zambia; 6 Department of Biostatistics and Epidemiology, School of Public Health, University of Zambia, Lusaka, Zambia; 7 Department of Paediatrics, Intensive Care Unit, Windhoek Central Hospital, Windhoek, Namibia; Istanbul University-Cerrahpasa, Cerrahpasa Medical Faculty, TURKEY

## Abstract

**Background:**

Whilst malaria is a prominent aetiology associated with acute kidney injury (AKI) in many parts of Africa, a shift in the traditional AKI aetiologies has been witnessed in sections of the continent. Additionally, limited access to dialysis worsens patient outcomes in these low-resource settings. This retrospective cross-sectional study aimed to determine the associated aetiologies, predictors of need for dialysis and malaria-associated AKI (MAKI), and outcomes of AKI and dialysis among children evaluated by the renal service in Lusaka, Zambia.

**Methods:**

The study sampled all children aged 16 years or below, diagnosed with AKI between 2017 and 2021, by the renal unit at the University Teaching Hospitals- Children’s Hospital (UTH-CH), and retrospectively abstracted their records for exposures and outcomes. AKI was defined using the Kidney Disease Improving Global Outcomes (KDIGO) 2012 criteria. Frequency and percentage distributions were used to describe the occurrence of AKI aetiologies and treatment outcomes. Predictors of the need for dialysis, MAKI, and poor treatment outcome were identified by using multivariable logistic regression models.

**Results:**

A total of 126 children diagnosed with AKI were included in this study. Malaria was the most frequent aetiology of AKI(61.1% (77/126, 95% Confidence Interval (CI): 52.0%-69.7%)). Of the 126 children with AKI, 74.6% (94) underwent dialysis. Predictors of the need for dialysis were oliguria (p = 0.0024; Odds ratio (OR) = 7.5, 95% CI: 2.1–27.7) and anuria (p = 0.0211; OR = 6.4, 95% CI = 1.3, 30.7). A fifth (18.3%, 23/126) of the children developed chronic kidney disease (CKD), 5.6% (7/126) died and, a year later, 77% (97/126) were lost to follow-up.

**Conclusion:**

At UTH-CH, malaria is the most frequent aetiology among children with AKI undergoing dialysis and children from low-medium malaria incidence areas are at risk; a considerable proportion of children with AKI need dialysis and Tenchoff catheter use in AKI is advocated.

## Introduction

The link between the acute kidney injury (AKI) syndrome and adverse clinical outcomes is well established [1–4]. Reports on the outcomes of paediatric AKI in Africa are scanty [5–8]. A recent systematic review pooled results of 41 studies on AKI from Africa and included 1937 paediatric patients who had a reported mortality of 34%, higher than the mortality of 13.8% reported in a meta-analysis that collated worldwide AKI data [5, 9]. In low-resource settings, it is recognised that the limited ability to provide organ support including renal replacement therapy (RRT), the lack of laboratory support, and limited access to health facilities may further contribute to this high mortality [7, 9–11].

Most aetiologies responsible for AKI in Africa are community-acquired [7, 9]. Aetiologies such as sepsis, malaria, and acute fluid loss due to gastroenteritis feature prominently [6, 7, 12]. AKI aetiologies have been known to change in response to evolution in medical practice, medical procedures and urbanisation as has occurred in many high-income countries (HIC), where a noted shift in aetiologies to multifactorial causes of AKI, chiefly in patients admitted to the intensive care unit (ICU), has been observed [3, 13, 14]. Olowu *et al*. in a systematic review on AKI in the African population reported the leading aetiologies of AKI as being sepsis (23%) and glomerular diseases (21%) while malaria caused only 12.1% of AKI in children. They included studies conducted between 1990 and 2014 but they did not analyse time-associated trends in aetiology. An earlier study conducted among hospitalized children in Nigeria reported a shift in the predominant AKI aetiology to sepsis in contrast to previous reports from that region [15]. There is a need for more epidemiological data on AKI from Africa as the importance of the various aetiologies generally attributed to tropical AKI tend to differ from region to region and from one hospital sub-population to another [6, 12–14]

These trends in the aetiology of AKI have not been observed across the whole continent. Findings from Malawi and the Democratic Republic of Congo (DRC) indicated that malaria remains the predominant cause of AKI and accounts for 84.4% and 40% of AKI among hospitalized paediatric patients respectively [16, 17]. Furthermore, studies in Ugandan children have shown that AKI in malaria occurs more frequently than was previously reported especially in patients with severe forms of malaria and that it worsens both short-term and long-term patient outcomes [11, 18, 19]. The increased reports of MAKI may be the result of the widespread adoption of newer AKI diagnostic criteria, which are more sensitive [11, 20]. The rise in cases may also represent altered immunopathology because of successful malaria control programmes [21].

Although the exact pathophysiology and predictors of MAKI have not been completely elucidated, it is known that factors such as dehydration increase the risk of renal dysfunction associated with malaria-induced intravascular haemolysis [21]. In addition, factors that increase red cell haemolysis such as parasite drug resistance, a high parasite load and delayed presentation to a health facility for treatment may increase the risk of MAKI [22, 23]. Children with Glucose-6-phosphate dehydrogenase deficiency (G6PDH), an X-linked recessive disorder, have reduced levels of NADPH which leads to haemolysis when red cells are exposed to drug or infection-associated oxidative stress. In the context of malaria, patients with G6PDH deficiency have a higher susceptibility to severe haemolysis and may thus be at increased risk of haeme-associated AKI [24, 25] Finally, the host immunological response and endothelial activation influence the severity of parasite sequestration, which may in turn compromise renal perfusion [22, 26]. The interplay between all these factors and the variation in their expression in different populations may explain the disparities in the prevalence of MAKI.

This retrospective study aimed to determine the prevalence, aetiology, and outcomes of AKI among patients seen during the five years since the nephrology unit was established. Furthermore, we sought to determine the proportion and predictor characteristics of AKI patients that required dialysis as well as determine the complications associated with the use of RRT in our cohort of patients. The data for the first time provide some baseline information on AKI at a referral paediatric nephrology unit in Zambia and includes data on a sizable cohort of children with stage 3 MAKI.

## Materials and methods

### Study design, setting and population

This was a retrospective analytical cross-sectional study aimed at determining the aetiologies, predictors of dialysis and outcomes among AKI patients at the University Teaching Hospitals-Children’s Hospital (UTH-CH), nephrology service, in Lusaka, Zambia. Additionally, predictors of MAKI in AKI patients were determined. The UTH-CH is a 350-bed tertiary-level hospital and houses one of the two centres offering paediatric dialysis in Zambia. The target population for the study was patients less than 16 years of age, seen and followed up by the paediatric nephrology unit.

Most of the children with AKI are cared for in a 9-bed nephrology ward which has a two-bed haemodialysis unit ensuite and which has access to plasmapheresis facilities. Children considered in need of multiple organ support receive initial care in the 8- bed level II PICU.

Conventional peritoneal dialysis (PD) is employed, largely using modified adult Tenchoff catheters and standard dialysis fluids. However, when these conventional consumables are unavailable, both improvised catheters (Naso-gastric tubes (NGT)) and fluids are utilized.

Improvised fluids and catheters are prepared as previously described [27]. However, since 3-way taps are unavailable the improvised catheter is connected to the conventional PD Y- system directly. The improvised catheters are inserted by surgeons at the bedside while Tenchoff catheters are inserted at the bedside by either the surgical team or the nephrologist.

### Inclusion and exclusion criteria

Included were all patients identified as having AKI, aged 16 years old or below, at UTH-CH, seen and followed up by the paediatric nephrology unit, from February 2017 to December 2021. The study data were collected between September 2020 to August 2020 and then June-July 2022. This break was necessitated by COVID-19 waves. Excluded were children seen by the nephrology service but followed up by general paediatric units; excluded because of unavailable clinical records. Additionally, during the COVID-19 outbreaks, all patients and parents coming into the hospital were tested for covid using a rapid antigen test followed by PCR for confirmation. All covid patients were cared for in an isolation ward or at the designated covid centre and thus no covid patients were included in this cohort.

### Study sample

From February 2017 to December 2021, 410 children aged 16 years or below, at UTH-CH, were evaluated for renal disorders. All patients who were diagnosed with AKI and followed up by the paediatric nephrology Unit, made the sample for this study.

### Data collection

A structured form for abstracting data from patient records at the UTH-CH paediatric nephrology was designed. The form consisted of questions on demographic and baseline clinical features, laboratory, histopathological characteristics, and patient outcomes. All files in the renal registry were screened to identify children diagnosed with AKI. The form was administered to all the files for AKI children meeting the inclusion criteria.

### The data collected

The data collected comprised both dependent and independent variables for the study. Each recruited patient was assigned a unique study number which was used on the pre-designed data collection form and during data analysis.

### Dependent variables

The data collected consisted of whether the AKI patient was dialyzed or not, as a dependent variable, whether an AKI patient had malaria or not and patient outcomes as other dependent variables.

The outcomes assessed in this study were renal function at the time of discharge from the ward, the length of hospital stay, the renal outcome a year after the AKI episode, and known patient outcome at last contact with the patient.

Known patient outcome was further categorised into good outcomes and poor outcomes. Good treatment outcome meant the patient had ‘normal kidney function’ or ‘resolving kidney function’ at the last contact. Poor treatment outcome meant the patient had ‘absconded’, had ‘chronic kidney disease (CKD)’, had received ‘palliation’ or had ‘died’ as at the last contact.

### Independent variables

Variables considered as independent were classed under socio-demographic and clinical variable categories.

### Socio-demographic independent variables

Socio-demographic independent variables included the patient’s sex, age, referring hospital, province of origin, weight, and height.

### Clinical independent variables

Clinical independent variables included reasons for referral to the tertiary, aetiologies of AKI, and admission and peak creatinine and the estimated glomerular filtration rate (GFR) (calculated using the Schwartz formula (where patient height was unavailable, WHO centile charts were used to obtain the median height for sex and age in completed years [28].

Other clinical independent variables collected were the patient blood pressure at admission, presence of anuria or oliguria on admission, full blood count at admission, results of hepatitis infection screen and autoimmune screen and the HIV test result, the Rapid malaria antigen screen or parasite slide result.

Also noted were the patient’s recent drug history before to diagnosis of AKI, family history of renal disease, birth weight, duration of illness before to admission to UTH-CH, presence of haematuria (gross or microscopic), presence of proteinuria and grade on urinalysis, presence of skin rash/arthralgia or joint swelling during course of illness.

Additional independent clinical variables included were results of imaging tests (chest x-ray, renal ultrasound), indication for dialysis, medical cadre placing the PD catheter, ward where the catheter was inserted from, and any complications associated with the use of PD catheter.

### Data management

Ten percent of the completed data collection forms were randomly picked, and their entries checked against the patient files from which the data were abstracted. A level of concordance of less than 95% between the sampled data collection forms and corresponding patient files (mismatch rate >5%) would have meant a review for mismatches of all completed data collection forms against the patient files. However, only 3% of the forms were found with mismatches, and were corrected.

The data collection forms were entered into an IBM ^TM^-SPSS ^TM^ version 25 (IBM Corp., Armonk, NY, USA) database, and initial data cleaning and exploratory analyses were run using this software. The database was maintained on a password-protected computer and only shared with the core study team. The database was converted to a SAS ^®^ 9.4 (SAS Institute Inc., Cary, NC, USA) database for further cleaning and statistical analyses. Further cleaning involved running codes on the data to detect out-of- range, and incorrect data values. Anomalies were verified and corrected against patient records. Complete case analysis was the approach used in the presence of missing values.

### Statistical analyses

Statistical analyses involved determining descriptive statistics of all the variables and building statistical models to determine the predictors of dependent variables.

### Descriptive statistics

The database encompassed both continuous and discrete variables. The descriptive statistics determined for normally distributed continuous variables were their means along with standard deviations. Normality was checked using the Shapiro-Wilk test. The descriptive statistics determined for non-normally distributed continuous variables were their medians along with interquartile ranges. Discrete variables were described using their frequency and percentage distributions. Binomial distribution based on exact 95% CI was included in the frequency and percentage distribution of aetiologies, types of dialysis and peritoneal dialysis catheter indications.

### Models building

All the variables considered dependent in this study had binary responses. As such, their predictors were determined using logistic regression models. The models were built through two-stage processes. The first stage involved assessing bi-variable significance of associations between potential predictors and the dependent variable. The second stage involved building multivariable logistic regression models in a forward-step process. The details of the logistic regression model building process are provided in **S1 Appendix.**

### Ethical considerations

Since this is a record review, a waiver of consent was sought and received from the University of Zambia Biomedical Research Ethics Committee (UNZABREC REF. No. 406–2019). During data collection the authors had access to patient identifiers but to protect patient confidentiality data were anonymized for data analysis to protect patient confidentiality. Further clearance was obtained from the National Health Research Authority (NHRA).

## Results

### Recruitment of study subjects

**Fig 1:**
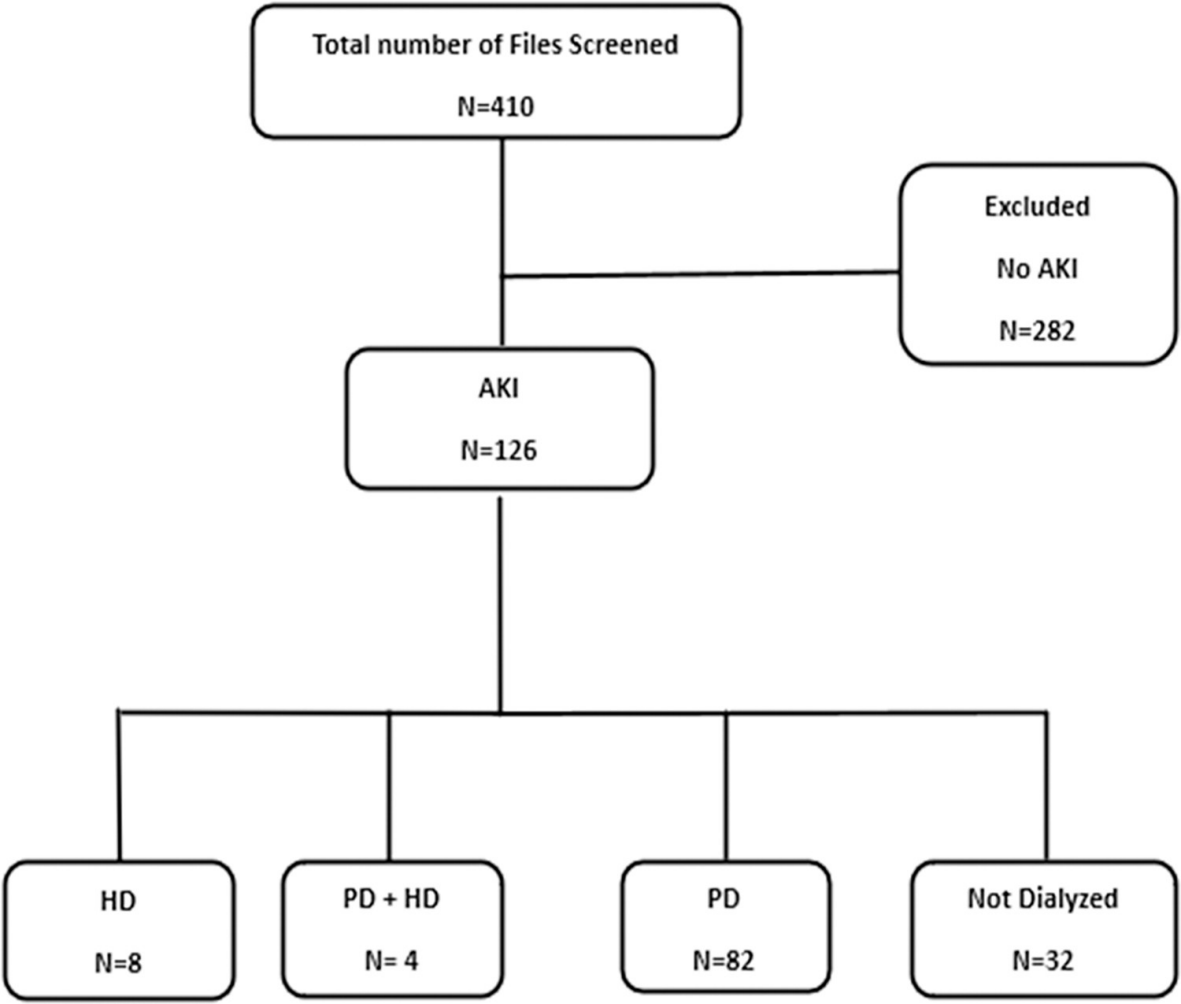
Study algorithm showing the recruitment of AKI patients from University Teaching Hospitals-Children’s Hospital, nephrology service. AKI = Acute kidney injury, HD = Haemodialysis, PD = Peritoneal dialysis.

Fig 1 summarises the recruitment algorithm for this study. In total 410 files of children evaluated by the nephrology service in the period under review were screened. Of these children 126 (37.7% of 410) had AKI. Eighty-two (65.1% of 126) of the AKI patients underwent peritoneal dialysis while only 8 (6.3% of 126) underwent haemodialysis, 4 (3.1% of 126) underwent both PD and HD, and 32 (25.4% of 126) were managed conservatively.

### Socio-demographic characteristics of study participants

Demographic characteristics of the recruited children for the study are presented in **Table 1**. Most of the children were boys (57.9%, 73/126) and the children’s median age was 7.5 years (yrs.) (interquartile range (IQR): 5 yrs.-11 yrs.). The majority of the children were older than 5 years of age (70.6%, 89/126). Most of the children were referred from provinces other than Lusaka (62.7%, 79/126). The children were of mean height 118.2 centimetres (cm) (standard deviation (SD): 22.6 cm) and median weight 21.3 kilogrammes (kg) (IQR: 16.7 kg-28.2 kg).

**Table 1:** Socio-demographic characteristics of patients in the sample by not dialyzed and dialyzed.

		Total	NOT DIALYSED	DIAYSED	
Characteristic	Category	(N = 126)	(N = 77)	(N = 49)	P-Value
**Sex**	n (%) Male	73 (57.9)	23 (71.9)	50 (53.2)	0.101*
	n (%) Female	53 (42.1)	9 (28.1)	44 (46.8)	
**Age (years)**	n (%) < 1 year	3 (2.4)	1 (3.1)	2 (2.1)	0.539
	n (%) 1 to < 5 Years	34 (27.0)	7 (21.8)	27 (28.7)	
	n (%) 5 to < 10 years	49 (38.9)	11 (34.4)	38 (40.4)	
	n (%) >10 years	40 (31.7)	13 (40.6)	27 (28.7)	
**Age (years)**	Median	7.5	8.0	7.0	0.418
	Interquartile range	5.0–11.0	5.1–12.0	5.0–11.0	
	N	126	32	94	
**Referring Province**	n (%) Outside Lusaka	79 (62.7)	16 (50.0)	63 (67.7)	0.114*
	n (%) Lusaka	46 (36.5)	16 (50.0)	30 (32.3)	
**Height (cm)**	Mean	118.2	115.7	119.0	0.657
	SD	22.6	24.7	22.1	
	N	57	18	5	
**Weight (kg)**	Median	21.3	22.1	21.0	0.491
	Interquartile range	16.7–28.2	17.2–32.3	15.5–28.0	
	N	124	32	92	

*p</ = 0.25, variable included in construction of multiple logistic regression model

SD stands for Standard Deviation. Some column percentages are not adding up to exactly 100% because of rounding off.

### Patient baseline clinical characteristics

Patient baseline clinical characteristics are presented in **Table 2**. The median illness duration prior to presentation was 7.0 (IQR 5.0–14.0) days and illness duration were not significantly different between children that received dialysis and those that did not. Oedema was the most common clinical presentation (65.9%,83/126) among the children.

**Table 2:** Participant clinical characteristics by dialysis status.

		Total	NOT DIALYSED	DIAYSED	P-Value
Characteristic	Category	(N = 126)	(N = 77)	(N = 49)	
**Duration of illness before presentation in days**	Median	7.0	7.0	8.0	0.239*
	Interquartile range	5.0–14.0	4.5–13.0	5.0–14.0	
	N	121	32	89	
**Season**	n (%) 1^st^ Quarter	45 (35.7)	9 (7.1)	36 (28.6)	0.330
	n (%) 2^nd^ Quarter	46 (36.5)	10 (7.9)	36 (36.5)	
	n (%) 3^rd^ Quarter	14 (11.1)	5 (4.0)	9 (7.1)	
	n (%) 4^th^ Quarter	21 (16.7)	8 (6.4)	13 (10.3)	
**Hypertension**	n (%) Yes	41 (33.1)	9 (28.1)	32 (34.8)	0.637
	n (%) No	83 (66.9)	23 (71.9)	60 (65.2)	
**Oedema**	n (%) Yes	83 (65.9)	15 (46.9)	68 (72.3)	0.016*
	n (%) No	43 (34.1)	17 (53.1)	26 (27.7)	
**Gross**	n (%) Yes	23 (18.3)	11 (34.4)	12 (12.8)	0.014*
**Haematuria**	n (%) No	103 (81.7)	21 (65.6)	82 (87.2)	
**Anuria**	n (%) Yes	37 (29.4)	4 (12.5)	33 (35.1)	0.028*
	n (%) No	89 (70.6)	28 (87.5)	61 (64.1)	
**Oliguria**	n (%) Yes	48 (38.1)	5 (15.6)	43 (45.7)	0.005*
	n (%) No	78 (61.9)	27 (84.4)	51 (54.3)	
**Fever**	n (%) Yes	45 (35.7)	14 (43.8)	31 (33.0)	0.376
	n (%) No	81 (64.3)	18 (56.3)	63 (67.0)	
**Seizure**	n (%) Yes	22 (17.5)	3 (9.4)	19 (20.2)	0.261
	n (%) No	104 (82.5)	29 (90.6)	75 (79.8)	
**Reduced**	n (%) Yes	16 (12.7)	2 (6.3)	14 (14.9)	0.355
**GCS**	n (%) No	110 (87.3)	30 (93.8)	80 (85.1)	
**HIV**	n (%) Yes	7 (5.6)	3 (9.4)	4 (4.3)	0.369
	n (%) No	119 (94.4)	29 (90.6)	90 (95.7)	
**Hepatitis B Surface Antigen positive**	n (%) Yes	0 (0.0)	0 (0.0)	0(0.0)	0.837
	n(%) No	55 (44)	13 (41.9)	42 (44.7)	
	n(%) Not done	70 (56)	18 (58.1)	52 (55.3)	
**Hepatitis C**	n(%) Yes	0 (0.0)	0 (0.0)	0 (0.0)	1.000
	n(%) No	31 (24.8)	8 (25.8)	23 (24.5)	
	n(%) Not done	94 (75.2)	23 (74.2)	71 (75.5)	
**Syphilis (RPR)**	n(%) Yes	0 (0.0)	0 (0.0)	0 (0.0)	0.497
	n(%) No	35 (28.0)	7 (22.6)	28 (29.8)	
	n(%) Not done	90 (72.0)	24 (77.4)	66 (70.2)	
**Mechanical**	n (%) Yes	2 (1.6)	0 (0.00)	2 (2.1)	1.000
**Ventilation**	n (%) No	124 (98.4)	32 (100.0)	92 (97.9)	
**Reduced corticomedullary differentiation (KUBUS)**	n (%) Yes	5 (5.4)	25 (100.0)	63 (92.7)	0.319
	n (%) No	88 (94.6)	0 (0.0)	5 (7.4)	
**Echogenicity (KUBUS)**	n(%) Normal	63 (50.8)	16 (50)	47 (52.2)	0.918
	n(%) Increased	31 (25.0)	9 (28.1)	22 (24.4)	
	n(%) Not done)	28 (22.6)	7 (21.9)	21 (23.3)	
**Hydronephrosis**	n(%)Yes	12 (9.6)	3 (9.4)	9 (9.7)	0.503
	n(%)No	97 (77.6)	23 (71.9)	74 (79.6)	
	Not done	16 (12.8)	6 (18.8)	10 (10.8)	
**Admission Haemoglobin**	Median	7.0	7.8	6.8	0.228*
	Interquartile range	5.9–9.1	6.4–9.5	5.7–8.9	
	N	122	31	91	
**White cell count**	Median	13.4	12.8	13.5	0.874
	Interquartile range	9.0–18.5	7.6–20.9	9.4–18.5	
	N	122	31	91	
**Admission platelet count**	Median	269.0	322.0	264.0	0.142*
	Interquartile range	115.0–465.0	138.0–584.0	106.0–437.0	
	N	122	31	91	
**Admission serum sodium**	Median	133.0	137.0	132.5	0.143*
	Interquartile range	128.0–138.0	132.0–138.0	127.0–139.0	
	N	77	19	58	
**Admission serum creatinine**	Median	758.0	563.0	883.0	<0.001*
	Interquartile range	489.0–1104.0	344.0–744.8	602.0–1190.0	
	N	126	32	94	
**Days to peak creatinine**	Median	1.0	0.0	2.0	0.088*
	Interquartile range	0.0–5.0	0.0–4.5	0.0–5.0	
	n	126	32	94	
**Admission KDIGO AKI stage**	Stage 1	1 (0.8)	1 (3.2)	0 (0.0)	0.272
	Stage 2	6 (4.8)	2 (6.5)	4 (4.3)	
	Stage 3	117 (84.9)	28 (90.3)	89 (95.7)	
**Peak creatinine**	Median	934.0	670.0	1036.5	<0.001*
	Interquartile range	658.0–1191.0	434.0–909.8	729.0–1324.0	
	N	126	32	94	
**Aetiology AKI**	n (%) Malaria	77 (61.1)	17 (53.1)	60 (63.8)	0.388
	n (%) non-Malaria	49 (38.9)	15 (46.9)	34 (36.2)	
**Aetiology Category**	n (%) Malaria	77 (61.1)	17 (53.1)	60 (63.8)	0.105*
	n (%) Glomerulonephritis	13 (10.3)	5 (15.6)	8 (8.5)	
	n (%) HUS	8 (6.4)	3 (9.4)	5 (5.3)	
	n (%) PUV	6 (4.8)	3 (9.4)	3 (3.2)	
	n (%) Sepsis	5 (4.0)	1 (3.1)	4 (4.3)	
	n (%) Hypovolaemia	3 (2.4)	2 (6.3)	1 (1.1)	
	n (%) Others	14 (11.1)	1 (3.1)	13 (13.8)	
**Outcome**	n (%) Bad	33 (26.2)	2 (6.3)	31 (33.0)	0.006*
	n (%) Good	93 (73.8)	30 (93.8)	63 (67.0)	

*p</ = 0.25, variable included in construction of multiple logistic regression model

IQR stands for Interquartile Range. GCS stands for Glasgow coma scale. RC-MD stands for Reduced Cortico-medullary Differentiation. KUBUS stands for kidney ureter blader ultrasound. HF stands for Health Facility. Some column percentages are not adding up to exactly 100.0% because of rounding off.

The vast majority of the children were diagnosed with AKI at the referring health facility (69.8% (88/126), while 3.2% (4/126) were referred for severe malaria, three for suspected chronic kidney disease (CKD), and two for glomerulonephritis.

None of the children who were screened for syphilis (35), hepatitis B (55) or hepatitis C (31) were reactive. Of the 126 children, 5.6% (7/126) had HIV.

Kidney ureter and bladder ultrasound (KUBUS) findings were recorded for 93 patients; 33.3% (31/93) had increased renal echogenicity and 5.4% (5/93) had reduced cortico-medullary differentiation. Of the recorded kidney sizes, 13 had increased kidney size, three had reduced kidney size and one had a discrepancy in size between the two kidneys. Hydronephrosis was reported in twelve children.

The multivariable logistic regression model for the prediction of dialysis among patients in the sample is shown in **Table 3**. Peak creatinine, days to peak creatinine, and a combined consideration of peak creatinine and days to peak creatinine were significant predictors of dialysis among the children; p-values equal to 0.002, 0.023 and 0.038 respectively. Other potential predictors of the need for dialysis found significant were anuria, oliguria at admission and platelets; P-values equal to 0.003, 0.004 and 0.044 respectively.

**Table 3:** Multivariable logistic regression model for prediction of dialysis among patients in the sample, N = 121.

Effect		Standard Error	Wald Chi-Square	Pr > ChiSq	Odds Ratios	95% Confidence Intervals of Odds Ratios	
						Lower Limit	Upper Limit
**Intercept**	-0.134	1.059	0.016	0.900			
**Peak creatinine**	0.003	0.001	9.361	0.002*	-	-	-
**Days to peak creatinine**	0.442	0.194	5.173	0.023*	-	-	-
**Peak creatinine and Days to peak creatinine**	-0.0004	0.0002	4.328	0.038*			
**Oliguria: Yes vs No**	0.994	0.345	8.288	0.004*	7.30	1.89	28.23
**Oedema: Yes vs No**	0.406	0.314	1.671	0.196	2.25	0.66	7.72
**Anuria: Yes vs No**	1.186	0.396	8.951	0.003*	10.72	2.27	50.67
**Sex: Male vs Female**	-0.58	0.307	3.569	0.059	0.31	0.09	1.05
**Platelets**	-0.003	0.001	4.059	0.044*	1.00	1.00	1.00

Model statistics

Testing Global Null Hypothesis: BETA = 0: Likelihood Ratio chi-square = 52.36, df = 8, p = < .0001

Hosmer and Lemeshow Goodness-of-Fit Test: Chi-square = 6.75, df = 8, p = 0.5638

C-statistic: 0.875

*p</ = 0.05, p or pr stands for probability and chisq stands for chi square, df stands for degrees of freedom.

The quality of the model was high. At least one of the potential predictors was significant (Likelihood ratio (LR) chi-square = 52.36, p <0.001). The model’s predicted values were close to the observed values (Hosmer and Lemeshow (H&L) Chi-square = 6.75, p = 0.562); and its ability to classify correctly between ‘dialyzed’ and ‘not dialyzed’ in its predictions was reasonably strong (c-statistic = 0.8753).

### Aetiology of AKI

The recorded aetiologies of AKI in the sample are displayed in **Table 4**. Malaria was the most (61.1%, 95% CI: 52.0%-69.7%) frequent aetiology of AKI among the children. while 11(8.7%) had glomerulonephritis and 2 (1.6%) had systemic lupus erythematosus as the underlying cause of AKI.

**Table 4:** Breakdown of aetiologies of AKI, N = 126.

Aetiology	Frequency (Percent)	95% Confidence interval of percent
**Malaria**	77 (61.1)	52.0–69.7
**Glomerulonephritis**	11 (8.7)	4.4–15.1
**Haemolytic Uremic Syndrome**	8 (6.3)	2.8–12.1
**Posterior Urethral valves**	6 (4.8)	1.8–10.1
**Sepsis**	5 (4.0)	1.3–9.0
**Hypovolaemia**	3 (2.4)	4.9–6.8
**Systemic Lupus erythematosus**	2 (1.6)	0.2–5.6
**Reno-vascular hypertension**	2 (1.6)	0.2–5.6
**Nephrolithiasis**	2 (1.6)	0.2–5.6
**Traumatic injury**	2 (1.6)	0.2–5.6
**Herbal intoxication**	1 (0.8)	0.0–4.4
**Malignancy**	2 (1.6)	0.2–5.6
**Ureteric stenosis (schistosomiasis)**	1 (0.8)	0.0–4.4
**Brake fluid poisoning (para-suicide)**	1 (0.8)	0.0–4.4
**Enalapril**	1 (0.8)	0.0–4.4
**Intravascular haemolysis**	1 (0.8)	0.0–4.4
**Unknown**	1 (0.8)	0.0–4.4

### Seasonality of AKI

**Fig 2:**
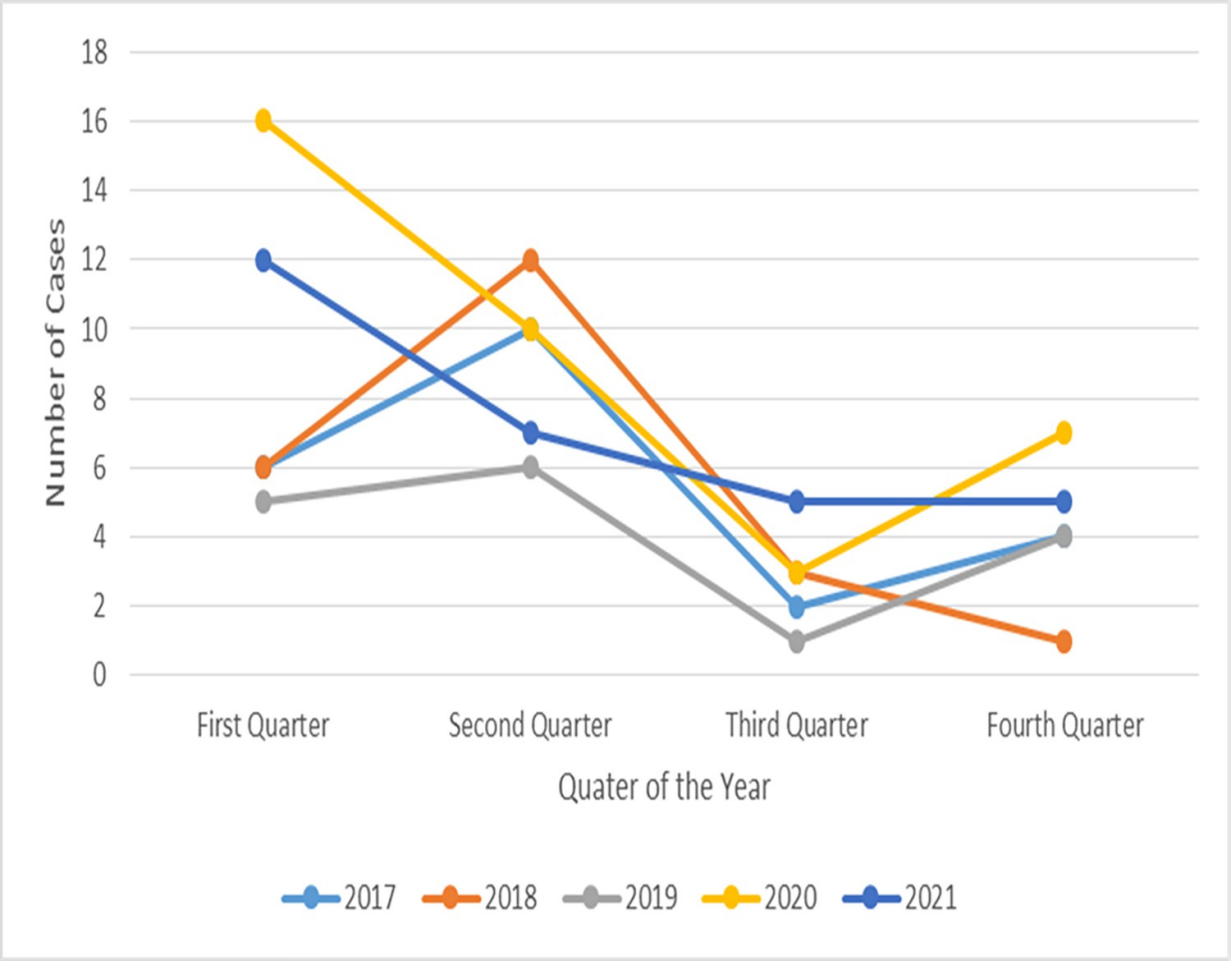
Time and seasonal trends in paediatric AKI cases presenting to UTH 2017–2021.

The time and seasonal trends in paediatric AKI cases presenting to UTH, from 2017 to 2021 are presented in **Fig 2**. There was marked seasonality observed in the number of AKI cases with most cases concentrated within the first six months of the year. The peak of AKI cases was observed in the second quarter in the years 2017, 2018, and 2019 but for the years 2020 and 2021 this peak in cases was not observed.

The first quarter coincides with the peak of the rainy season and the second quarter is the end of the rainy season into the beginning of the cool dry season. The third quarter is the cool and dry season whereas the fourth quarter is the hot dry season and the onset of the rainy season.

### Association of patient characteristics to MAKI

Distribution of patient characteristics when compared between children without malaria who had AKI caused by other aetiologies and children with MAKI is shown in **Table 5**. Children with MAKI were more likely to be older than 5 years compared to those with AKI caused by other aetiologies (p = 0.03). (Table 4) They were also more likely to be referred from outside Lusaka province (p = 0.08). MAKI patients were more likely to present with a shorter duration of illness (7.0 versus 7.5 days, p = 0.042). More MAKI patients had a reduced Glasgow coma scale (GCS), fever, and hypertension (p = 0.041, p = 0.0006, p<0.0001 respectively) at presentation than children with AKI caused by other aetiologies. MAKI patients had lower admission haemoglobin and platelet counts than did children with AKI caused by other aetiologies, but the admission creatinine was higher (869.0, IQR 542.0–1158.0 versus 646.0, IQR 542.0–1158.0, p = 0.054). MAKI patients were followed up for a shorter duration compared to patients with AKI caused by other aetiologies.

**Table 5:** Distribution of patient characteristics by other AKI aetiologies and MAKI.

Characteristic	Category	Total	Other AKI aetiologies	MAKI	P-Value
		(N = 126)	(N = 49)	(N = 77)	
**Sex**	n (%) Male	73 (57.9)	26 (53.1)	47 (61.0)	0.484
	n (%) Female	53 (42.1)	23 (46.9)	30 (39.0)	
**Age (years)**	Median	7.5	8.0	8.2	0.912
	Interquartile range	5.0–11.0	4.0–12.0	5.0–11.0	
	N	126	49	77	
** Age categorized (years)**	n (%) < 1 year	3 (2.4)	3 (6.1)	0 (0.0)	0.025*
	n (%) 1 to < 5 Years	34 (27)	14 (28.6)	20 (26.0)	
	n (%) 5 to < 10 years	49 (38.9)	13 (26.5)	36 (46.8)	
	n (%) >10 years	40 (31.7)	19 (38.8)	21 (27.3)	
**Referring Province**	n (%) Outside Lusaka	79 (62.7)	26 (53.1)	53 (69.7)	0.087*
	n (%) Lusaka	46 (36.5)	23 (46.9)	23 (30.3)	
**Height**	Mean	118.2	121.8	115.9	0.385
	SD	22.6	27.1	19.4	
	N	57	22	35	
**Weight**	Median	21.3	23.3	21	0.992
	Interquartile range	16.7–28.2	14.0–35.0	17.4–26.0	
	N	124	49	75	
**Duration of illness before presentation**	Median	7.0	7.5	7.0	0.043*
	Interquartile range	5.0–14.0	6.0–21.0	4.0–14.0	
	N	121	48	73	
**Season**	n (%) 1^st^ Quarter	45 (35.7)	15 (30.6)	30 (39.0)	0.006*
	n (%) 2^nd^ Quarter	46 (36.5)	13 (26.5)	33 (42.9)	
	n (%) 3^rd^ Quarter	14 (11.1)	11 (22.5)	3 (3.9)	
	n (%) 4^th^ Quarter	21 (16.7)	10 (20.4)	11 (14.3)	
**Oedema**	n (%) Yes	83 (65.9)	34 (69.4)	49 (63.6)	0.638
	n (%) No	43 (34.1)	15 (30.6)	28 (36.4)	
**Macroscopic Haematuria**	n (%) Yes	23 (18.3)	6 (12.2)	17 (22.1)	0.164
	n (%) No	103 (81.7)	43 (87.8)	60 (77.9)	
**Anuria**	n (%) Yes	37 (29.4)	14 (28.6)	23 (29.9)	1.000
	n (%) No	89 (70.6)	35 (71.4)	54 (70.1)	
**Oliguria**	n (%) Yes	48(38.1)	17 (34.7)	31 (40.3)	0.661
	n (%) No	78 (61.9)	32 (65.3)	46 (59.7)	
**Impaired consciousness**	n (%) Yes	16 (12.7)	2 (4.1)	14 (18.2)	0.041*
	n (%) No	110 (87.3)	47 (95.9)	63 (81.8)	
**Fever**	n (%) Yes	45 (35.7)	8 (16.3)	37 (48.1)	0.001*
	n (%) No	81 (64.3)	41 (83.7)	40 (52.0)	
**Hypertension**	n (%) Yes	41 (33.1)	28 (57.1)	13 (17.3)	< .001*
	n (%) No	83 (66.9)	21 (42.9)	62 (82.7)	
**Admission Haemoglobin**	Median	7.0	7.2	6.7	0.024*
	Interquartile range	5.9–9.1	6.5–10.0	5.6–8.4	
	n	122	48	74	
**Admission white cell count**	Median	13.4	13.8	12.7	0.292
	Interquartile range	9.0–18.5	9.8–21.3	8.4–17.5	
	N	122	48	74	
**Admission platelet count**	Median	269.0	337.0	201.0	0.147*
	Interquartile range	115.0–465.0	216.0–524.5	74.0–434.0	
	N	122	48	74	
**Admission serum sodium**	Median	133.0	135.2	132.0	0.020*
	Interquartile range	128.0–138.0	130.0–141.0	127.0–137.0	
	N	77	35	49	
**Admission serum creatinine**	Median	459.8	646.0	869.0	0.054*
	Interquartile range	758.0–489.0	405.7–978.0	542.0–1158.0	
	N	126	49	77	
**Days to Peak Creatinine**	Median	1.0	0.0	2.0	0.205*
	Interquartile range	0.0–5.0	0.0–5.0	0.0–5.0	
	N	126	49	77	
**Peak creatinine**	Median	934.0	729.0	1035.0	0.007*
	Interquartile range	658.0–1191.0	594.0–1134.0	747.0–1336.0	
	N	126	49	77	
**Admission KDIGO Stage**	n (%) stage 1	1 (0.7)	0 (0.0)	1 (1.3)	0.558
	n (%) Stage 2	6 (4.8)	2 (4.1)	4 (5.2)	
	n (%) Stage 3	117 (92.9)	47 (95.9)	70 (0.9)	
	n (%) Normal	2 (1.4)	0 (0.0)	2 (2.6)	
**HIV**	n (%) Yes	7 (5.6)	2 (4.1)	5 (6.5)	0.705
	n (%) No	119 (94.4)	47 (95.9)	72 (93.5)	
**Dialysed**	n (%) Yes	94 (74.6)	34 (69.4)	60 (77.9)	0.301
	n (%) No	32 (25.4)	15 (30.6)	17 (22.1)	
**Outcome**	n (%) Bad	33 (26.2)	16 (32.7)	17 (22.1)	0.216*
	n (%) Good	93 (73.8)	33 (67.4)	60 (77.9)	
**Patient outcome at Discharge from hospital**	n (%) Palliation	10 (7.9)	5 (10.2)	5 (6.5)	0.113*
	n (%) Dialysis free	106 (84.1)	39 (79.6)	67 (87.0)	
	n (%) Home PD	3 (2.38)	0 (0.0)	3 (3.9)	
	n (%) Death	3 (2.38)	3 (6.1)	0 (0.0)	
	n (%) LAMA	4 (3.2)	2 (4.1)	2 (2.6)	
**Duration follow-up**	Median	33.5	45.0	28.0	0.095*
**(days)**	Interquartile range	18.0–94.0	23.0–120.0	17.0–63.0	
** **	N	126	49	77	

* p-value </ = 0.25, variable included in construction of multiple logistic regression model

Total column percentage may not total up to exactly 100.0% because of rounding up percentages to one decimal place. LAMA stands for left against medical advice, PD stands for peritoneal dialysis.

### Geographical distribution of the MAKI cases

When the cases of MAKI were mapped in comparison to the malaria transmission map of Zambia, it was found that most cases originated from the southern half of the country, which is classified as a low-medium transmission malaria zone.

### Predictors of MAKI

The multivariable logistic regression model for prediction of MAKI among patients in the sample is shown in **Table 6**. In this model, the potential predictors of MAKI found significant were fever, hypertension, and sodium; P-values 0.026, 0.008, and 0.012 respectively.

**Table 6:** Multivariable logistic regression model for prediction of MAKI among patients in the sample, N = 71.

						95% Confidence Intervals of Odds Ratios	
Effect	Estimate	Standard Error	Wald Chi-Square	Pr > ChiSq	Odds Ratios	Lower Limit	Upper Limit
**Intercept**	18.172	6.761	7.225	0.007	-	-	-
**Fever: Yes vs No**	0.741	0.333	4.954	0.026*	4.40	1.19	16.22
**Hypertension: Yes vs No**	-0.877	0.328	7.160	0.008*	0.17	0.05	0.63
**Haemoglobin**	-0.283	0.142	3.952	0.047*	0.75	0.57	1.00
**Sodium**	-0.121	0.048	6.371	0.012*	0.89	0.81	0.97

Model statistics

Testing Global Null Hypothesis: BETA = 0: Likelihood Ratio chi-square = 29.71, df = 4, p = < .001

Hosmer and Lemeshow Goodness-of-Fit Test: chi-square = 10.65, df = 8, p = 0.222

C-statistic: 0.833

*P-value </ = 0.05, p or pr stands for probability and chisq stands for chi square, df stands for degrees of freedom

The quality of the model was very high; at least one of the potential predictors was significant (LR chi-square = 29.71, p<0.001); the model’s predicted values were close to the observed values (H&L chi-square = 10.65, p = 0.222); and its ability to classify correctly between ‘MAKI’ and ‘not MAKI’ in its predictions was reasonably strong (c-statistic = 0.833).

### Dialysis outcomes

Types of dialysis and peritoneal dialysis (PD) catheter indications are shown in **Table 7**. The majority of all the AKI patients underwent dialysis (74.6% (94/126)). The most utilized dialysis modality was PD at 87.2% (82/94). These 82 children who received PD utilized a total number of 147 PD catheters because some of the catheters had to be replaced due to complications such as blockage. The leading clinical indications for the insertion of PD catheter were fluid overload in 34.6% (51/147) and hyperkalaemia in 14.3% (21/147).

**Table 7:** Type of dialysis and peritoneal dialysis catheter indications, N = 147.

Characteristic		Frequency (Percent)	95% Confidence interval of percent
**Type of dialysis done:**			
	Haemodialysis	8 (8.5)	3.8–16.1
	Peritoneal dialysis	82 (87.2)	78.8–93.2
	Both Haemodialysis and Peritoneal dialysis	4 (4.3)	1.2–10.5
**Peritoneal dialysis Catheter Indications: N = 147**			
	Acidosis	10 (4.0)	2.7–10.1
	Blood products	16 (9.0)	1.6–8.0
	Catheter block	58 (32.8)	5.3–14.3
	Uraemia	11 (6.2)	3.1–10.9
	Fluid overload	51 (28.8)	26.0–40.2
	Hyperkalaemia	21 (11.9)	22.3–36.1
	MODs	3 (1.7)	7.5–17.6
	Anuria	7 (4.0)	0.4–4.9

An average of 1.8 catheters were inserted per patient per AKI episode. Characteristics of PD catheter use by type of catheter (Tenchoff versus improvised) are shown in **Table 8**. Out of the 147 catheters inserted, 73.8% (104/147) were associated with the occurrence of a complication. There was no difference in the overall complication rate between Tenchoff catheters and improvised catheters (58 versus 51, p = 0.25). However, omental block, leak and primary non-function were more common when improvised catheters were used. In contrast peritonitis occurred more frequently in association with Tenchoff catheter use (34 versus 17, p = 0.07).

**Table 8:** Characteristics of peritoneal dialysis catheter use by type of catheter (Tenchoff versus improvised), N = 147.

Characteristic	Category	Total	Tenchoff Dual Cuff Catheter	Improvised (Naso-gastric tube) Catheter	P- Value
		(N = 147)	(N = 83)	(N = 64)	
**Who inserted**	n (%) Surgical Reg.	111 (75.5)	50 (60.2)	61 (95.3)	< .001*
**PD Catheter**	n (%) Nephrologist	30 (20.4)	30 (36.1)	0 (0.0)	
** Where was insertion done?**	n (%) Surgeon	6 (4.1)	3 (3.6)	3 (4.7)	0.011*
	n (%) Renal Ward	87 (59.2)	58 (69.9)	29 (46.0)	
	n (%) PICU	43 (29.3)	17 (20.5)	26 (41.3)	
	n (%) Theatre	12 (8.2)	5 (6.0)	7 (11.1)	
	n (%) Emergency room	4 (2.8)	3 (3.6)	1 (1.6)	
**Duration (Days) Catheter Used**	Median	9.0	12.0	5.0	< .001*
	Interquartile range	3.0–15.0	(5.0–23.0	1.0–9.0	
	N	147	83	64	
**Complication**	n (%) Yes	104 (73.8)	58 (69.9)	51 (79.7)	0.247
	n (%) No	37 (26.2)	25 (30.1)	13 (20.3)	
**Peritonitis**	n (%) Yes	51 (34.7)	34 (41.0)	17 (26.6)	0.100*
	n (%) No	96 (65.3)	49 (59.0)	47 (73.4)	
**Omentum/fibrin Block**	n (%) Yes	56 (38.1)	21 (25.3)	35 (54.7)	0.001*
	n (%) No	91 (61.9)	62 (74.7)	29 (45.3)	
**Leak**	n (%) Yes	9 (6.1)	1 (1.2)	8 (87.5)	0.011*
	n (%) No	138 (93.9)	82 (98.8)	56 (12.5)	
**Primary Non-function**	n (%) Yes	8 (5.4)	2 (2.4)	6 (9.4)	0.079
	n (%) No	139 (94.6)	81 (97.6)	58 (90.6)	
**Haemorrhagic Dialysate**	n (%) Yes	5 (3.4)	4 (4.8)	1 (1.6)	0.535
	n (%) No	142 (96.6)	79 (95.2)	63 (98.4)	
PD-associated Sepsis	n (%) Yes	4 (2.7)	1 (1.2)	3 (4.7)	0.318
	n (%) No	143 (97.3)	82 (98.8)	61 (95.3)	
**Wound infection**	n (%) Yes	2 (1.4)	1 (1.2)	1 (1.6)	1.000
	n (%) No	145 (98.6)	82 (98.8)	63 (98.4)	
**Dislodged Catheter**	n (%) Yes	1 (0.7)	1 (1.2)	0 (0.0)	1.000
	n (%) No	146 (99.3)	82 (98.8)	64 (100.0)	

* P-value </ = 0.05

PD stands for peritoneal dialysis.

### AKI patient outcomes

The outcomes assessed in this study were renal function at the time of discharge from the ward, length of hospital stay, renal function at last contact with the patient, the renal outcome a year after the AKI episode, and known patient outcome at last contact with the patient (**Table 9**).

**Table 9:** Patient Outcomes at discharge from the renal ward, N = 126.

Outcome	Condition		Total
		Frequency (Percent)	n (%)
**Good**	Normal Kidney Function	58 (46.0)	91(72.2)
	Resolving kidney function at last contact	33 (26.2)	
**Poor**	Absconded	7 (5.6)	35 (27.8)
	Chronic kidney disease	15 (11.9)	
	Palliation (ESRD)	10 (7.9)	
	Death	3 (2.3)	

The patient eGFR are compared to the outcome groups at the time of discharge from the ward in **Table 10**. During the 5 years under study, three children were commenced on home PD (Table10). Due to a shortage of consumables during the COVID-19 pandemic, all three children died.

**Table 10:** Patient glomerular filtration rate (GFR) at last contact versus treatment outcome group.

GFR at last Contact (ml/1.73m^2^ /ml)	Palliation	Dialysis Free	Home Dialysis	Death	Absconded	Frequency (Percent)
**>90**	0	58	0	0	0	58 (46.0)
**60–89**	0	1	0	0	1	2 (1.6)
**30–59**	0	14	0	0	0	14 (11.1)
**15–29**	0	22	0	0	1	23 (18.3)
**<15**	10	8	3	3	5	29 (23.0)

Of the 29 patients followed up for at least a year, twelve had a normal renal function, four had CKD stage 2–4 and one was on home PD. A total proportion of 77% (97/126) of AKI patients were followed up for less than 1 year. Of all the AKI patients, 34.1% (43/126) were transferred to other facilities for continued care while 42.9% (54/126) did not return for scheduled reviews and 9.5% (12/126) were known to have died (**Table 11).**

**Table 11:** Patient outcome at I year post-treatment.

Outcome	Frequency (Percent)
**Normal kidney function**	12 (9.5)
**CKD stage 2–4**	4 (3.2)
**ESRD on PD**	1 (0.8)
**Lost to follow-up**	54 (42.9)
**Transferred out to another facility**	43 (34.1)
**Death**	12 (9.5)
**Total**	126 (100)

### Predictors of poor patient outcome

Patient characteristics by outcomes at last contact with the renal service are shown in **Table 12**.

**Table 12:** Patient characteristics by outcomes at discharge from the renal ward, N = 126.

Characteristic	Category	Total	Outcome		P-Value
			Poor	Good	
		(N = 126)	(N = 33)	(N = 93)	
**Season**	n (%) 1^st^ Quarter	45 (35.7)	10 (30.3)	35 (37.6)	0.756
	n (%) 2^nd^ Quarter	46 (36.5)	13 (39.4)	33 (35.5)	
	n (%) 3^rd^ Quarter	14 (11.1)	5 (15.2)	9 (9.7)	
	n (%) 4^th^ Quarter	21 (16.7)	5 (15.2)	16 (17.2)	
Referred from Lusaka province	n (%) Outside Lusaka	79 (62.7)	25 (75.8)	54 (58.7)	0.125
	n (%) Lusaka	46 (36.5)	8 (24.2)	38 (41.3)	
**Age (years)**	Median	7.5	9.0	7.0	0.319
	Interquartile range	(5.0–11.0)	(5.0–12.0)	(5.0–11.0)	
	N	126	33	93	
**Age Category**	n (%) < 1 year	3 (2.4)	1 (3.0)	2 (2.2)	0.630
	n (%) 1 to < 5 Years	34 (27)	8 (24.2)	26 (28.0)	
	n (%) 5 to < 10 years	49 (38.9)	11 (33.3)	38 (40.9)	
	n (%) >10 years	40 (31.7)	13 (39.4)	27 (29.0)	
**Sex**	n (%) Male	73 (57.9)	17 (51.5)	56 (60.2)	0.506
	n (%) Female	53 (42.1)	16 (48.5)	37 (39.8)	
**Oedema**	n (%) Yes	83 (65.9)	7 (21.2)	36 (38.7)	0.108*
	n (%) No	43 (34.1)	26 (78.8)	57 (61.3)	
**Anuria**	n (%) Yes	37(29.4)	12 (36.5)	25 (26.9)	0.421
	n (%) No	89 (70.6)	21 (63.6)	68 (73.2)	
**Oliguria**	n (%) Yes	48(38.1)	15 (45.5)	33 (35.5)	0.421
	n (%) No	78 (61.9)	18 (54.6)	60 (64.5)	
**Impaired consciousness**	n (%) Yes	16 (12.7)	4 (12.2)	12 (12.9)	1.000
	n (%) No	110 (87.3)	29 (87.9)	81 (87.1)	
**Fever**	n (%) Yes	45 (35.7)	5 (15.2)	40 (31.8)	0.008*
	n (%) No	81 (64.3)	28 (84.9)	53 (57.0)	
**Hypertension**	n (%) Yes	41 (33.1)	16 (50.0)	25 (27.2)	0.032*
	n (%) No	83 (66.9)	16 (50.0)	67 (72.8)	
**Macroscopic Haematuria**	n (%) Yes	23(18.3)	2 (6.1)	21 (22.6)	0.037
	n (%) No	103(81.7)	31 (93.9)	72 (77.4)	
**HIV**	n (%) Yes	7 (5.6)	1 (3.0)	6 (6.5)	0.675
	n (%) No	119 (94.4)	32 (97.0)	87 (93.6)	
**Weight (kg)**	Median	21.3	25.3	20.9	0.0314*
	Interquartile range	16.7–28.2	20.3–32.2	15.6–26.1	
	N	124	33	91	
**Duration of illness before presentation**	Median	7.0	14.0	7.0	0.006*
	Interquartile range	5.0–14.0	7.0–21.0	5.0–14.0	
	N	121	32	89	
**Admission Haemoglobin**	Median	7.0	6.7	7.3	0.088*
	Interquartile range	5.9–9.1	5.6–7.5	6.0–9.6	
	N	122	32	90	
**Admission platelet count**	Median	269.0	319.5	265.0	0.352
	Interquartile range	115.0–465.0	151.0–465.5	87.0–465.0	
	N	122	32	90	
**Admission serum creatinine**	Median	758.0	904.8	685.0	0.004*
	Interquartile range	489.0–1104.0	665.0–1325.0	417.0–1045.0	
	N	126	33	93	
**Peak creatinine**	Median	934.0	1069.0	886.0	0.007*
	Interquartile range	658.0–1191.0	780.0–1642.0	631.0–1134.0	
	N	126	33	93	
**Malaria**	n (%) Yes	77 (38.9)	17 (51.5)	60 (64.5)	0.268
	n (%) No	49 (69.1)	16 (48.5)	33 (35.5)	

* P-value </ = 0.25, variable included in construction of multiple logistic regression model

Patient outcome was not correlated to the season in which they presented or the province from which they were referred.

The multivariable logistic regression model for prediction of poor treatment outcomes among patients in the sample is shown in **Table 13**. Fever, peak creatinine, and haemoglobin were found to be significant predictors of poor outcomes in AKI among children at UTH-CH; P-values 0.002, 0.006 and 0.033 respectively.

**Table 13:** Multiple logistic regression analysis of outcome classification (Poor vs Good), N = 120.

Effect	Estimate	Standard Error	Wald Chi-Square	Pr > ChiSq	Odds Ratios	95% Confidence Intervals of Odds Ratios	
						Lower Limit	Upper Limit
**Intercept**	-1.387	1.084	1.636	0.201	-	-	-
**Fever: Yes vs No**	-1.033	0.338	9.371	0.002*	0.13	0.03	0.48
**Peak creatinine**	0.002	0.001	7.598	0.006*	1.00	1.00	1.00
**Hypertension: Yes vs No**	0.475	0.260	3.338	0.068	2.59	0.93	7.18
**Haemoglobin**	-0.249	0.117	4.565	0.033*	0.78	0.62	0.98
**Oedema: Yes vs No**	0.412	0.291	2.003	0.157	2.28	0.73	7.13

Model statistics

Testing Global Null Hypothesis: BETA = 0: Likelihood Ratio chisq = 31.90, df = 5, p = < .001

Hosmer and Lemeshow Goodness-of-Fit Test: chisq = 9.23, df = 8, p = 0.322

C = 0.809

*P-value </ = 0.05 p or pr stands for probability and chisq stands for chi square, df stands for degrees of freedom

## Discussion

AKI cases constituted over a third of all patients followed up by the nephrology service. This is comparable to findings from a Ghanaian study in which AKI made up 31% of renal admissions over two years [29]. In contrast Obiagwu *et al*. in Kano Nigeria reported a prevalence of only 13.9% using KDIGO 2012 criteria [30]. The cohort from Kano consisted of only clinic attendees. As highlighted by Olowu *et al*. many African AKI cohorts report a high loss to follow-up rate so this may explain the lower prevalence reported in Kano as not all the AKI patients may have returned for a clinic review [9]. A study from northern Iran reported an even lower prevalence of only 7.3% but this study was conducted in 2004 before consensus AKI criteria were widely adopted [31].

There was a predominance of male patients in this cohort, similar to findings in other AKI datasets derived from Africa [7, 9, 15]. This pattern may be due to congenital anomalies of the kidney and urinary tract (CAKUT) which tend to occur more frequently in boys.

AKI patients had a median age of 7.5 (IQR 5.0–11.0) years and only three infants were included in this cohort. In contrast, studies conducted in Malawian, Ugandan and Nigerian hospitalised children with AKI, recruited significantly younger subjects with mean ages of 4 years, 1.7 years, and 4.8 years respectively [15, 19, 32]. Additionally, the present study showed no significant age difference between patients with MAKI and those without MAKI (p = 0.91). However, MAKI patients in the cohort were more likely to be older than 5 years of age. In contrast, Ugandan children with MAKI were much younger (1.7+/- 1.1 years) [17, 19]. But these studies deliberately recruited only children younger than 5 years of age. Nonetheless, historical reports of renal dysfunction in malaria seem to suggest that MAKI occurs more commonly in older children and non-immune adults from areas classified as zones of low malaria transmission [21, 33].

Most patients had severe AKI at admission. This is consistent with findings from earlier studies [7, 9]. One possible explanation could be the time it takes for patients in many low-resource countries(LIC) to get access to healthcare facilities [9]. Another reason could be that most of the children in this cohort had community-acquired acute kidney injury (CA-AKI) which was associated with a greater risk of severe AKI in some studies [34]. Also, this finding may be a mere reflection of selection bias resulting from the fact that the nephrology service is more likely to be consulted on children on the more severe end of the AKI spectrum. Data on the epidemiology of AKI among all hospitalized children is needed to better characterise this entity.

In contrast, a meta-analysis of worldwide pooled AKI data showed that only 11% of AKI patients had severe AKI [5]. The meta-analysis only included one study from Africa and the majority of patients in the meta-analysis had hospital-acquired acute kidney injury (HA- AKI). Studies have linked severe AKI to worse outcomes and increased risk of persistently abnormal creatinine [18, 35, 36]. It is thus important to identify strategies and interventions that can improve early diagnosis of AKI so that children are not put at increased risk of developing ESRD, which has a dire prognosis for many in LICs.

Most of the referring facilities were able to recognise AKI before sending the children to our hospital. It is known that AKI is often missed in hospitalised patients until it becomes severe or clinically apparent [7, 10, 37]. This may result in the late referral of patients and consequently may account for the high prevalence of severe AKI seen in many African cohorts [9]. There is need to improve the quality of epidemiological data on AKI from primary healthcare facilities to better characterise the predictors of MAKI development in children [38]. In addition, there is a need to train frontline workers on renal monitoring and initial non-dialytic management of children with suspected AKI especially the optimization of perfusion and avoidance of nephrotoxins [7, 10, 39–42].

Almost all (72, 93.5%) of the MAKI patients had received an anti-malarial drug before referral with less than 6 children exposed to quinine. This suggests that factors other than delayed access to anti-malarial medications may be responsible for the development of MAKI. In Zambia, treatment of malaria utilizes artemisinin-based therapies (artemether-lumefantrine and Dihydroartemisin-Piparaquine) as first line drugs in doses articulated in the national treatment guideline [43]. For severe malaria artesunate is recommended with quinine reserved as an alternative when artesunate is unavailable or contraindicated [43]. There are a few reports of renal dysfunction associated with use of artesunate, but it is largely considered safe in doses used in malaria treatment [44]. In fact, there are studies assessing artesunate for it’s anti-inflammatory effects to prevent AKI [45]. Artemether requires no renal adjustment in renal failure, but quinine may need adjustment [46]. Both Artemether and quinine do not require dose adjustment in patients on dialysis [46].

There was a marked seasonal distribution in the number of AKI cases with most of the cases concentrated in the first half of the year which is the rainy season (Fig 2). This seasonal variation in AKI may be attributable to seasonal changes in malaria cases which was the commonest aetiology of AKI in this cohort. It is known that there are variations in both sporozoite rates in the mosquito vector and in human infection rates that are related to seasonal changes in climatic conditions such as temperature and rainfall [47–50]. The second quarter peak of AKI cases was not observed in the years 2020 and 2021. This coincides with COVID-19 waves during the cool dry season in Zambia which were characterised by travel restrictions, and which could have influenced referral patterns and consequently the number of AKI cases seen in that period.

MAKI was the predominant aetiology identified. This contrasts with findings by Olowu *et a*l. who reported the leading aetiology of AKI in African children as septicaemia (22.5%) and showed that malaria accounted for only 12% of cases [9]. The findings from the current study demonstrate that in some regions of Africa, malaria is still the predominant aetiology of AKI contrary to what has been reported elsewhere [6, 11, 17, 19]. There is no universally adopted definition of MAKI. The WHO defines malaria associated AKI as the presence of malaria asexual forms and serum creatinine of Plasma or serum creatinine >265 μmol/l (3 mg/dl) or blood urea >20 mmol however, more recent consensus definitions of AKI such as the KDIGO 2012 AKI criteria, which was used in this study, have been found to be more sensitive [11, 20].

Additionally, although MAKI was identified as the leading cause of AKI in this cohort, no single test can confirm that AKI was solely due to malaria. There are no indications for renal biopsy that are specific to MAKI but a child with unresolving AKI beyond 14 days should probably undergo renal biopsy to rule out other aetiologies. No biopsies were performed on any of the MAKI patients included in this study and access to autoimmune screen tests was limited. In malaria-endemic areas some of these cases may merely represent asymptomatic malaria infection in children with AKI caused by different aetiologies.

In recent times there has been a noted increase in reports of AKI in children with malaria [11, 19, 21]. This may be the result of improved detection due to the adoption of newer AKI consensus guidelines [51]. In contrast, studies from West Africa in Ghanaian and Nigerian children report a very low prevalence of MAKI among children with malaria [29, 30]. This discrepancy in the importance of MAKI in different populations may be due to differences in biological or socio-economic factors [21, 26, 52]. In particular, differences in malaria transmission intensity may influence the pattern of disease manifestation including the incidence of MAKI [21, 52, 53]. It is known that change in malaria infection intensity because of malaria control programmes may lead to the occurrence of more severe disease in older people because the population is left with a higher proportion of non-immune individuals [52–54].

The MAKI patients in the present cohort were largely referred from medium transmission zones. Anecdotal information from the second paediatric dialysis centre in the northern region shows that most MAKI cases are from moderate transmission areas on the Copperbelt province with very few children referred for MAKI from high malaria transmission zones like Luapula, Muchinga and Northern provinces in the far north. There is a need for improved epidemiological mapping of AKI. Additionally, this data acts as a reminder to health workers that as the war on malaria is in the process of being completely won, malaria control programmes may leave sections of the population at increased risk of severe disease and altered manifestations of malaria. This calls for adjustments to control programmes to expand their target populations to these newer vulnerable sub-populations [53].

Predictors of MAKI were presence of fever, absence of hypertension at admission, and a higher admission serum creatinine (Table 5). Malaria often causes a catabolic type of AKI and many of the patients present with lower blood pressure as a result of dehydration caused by poor fluid intake and vomiting or due to systemic inflammatory response [21].

Patients were more likely to have MAKI if they had impaired consciousness at presentation (14 versus 2, p = 0.041). Children with severe forms of malaria such as cerebral malaria are known to be at higher risk of AKI [55]. Some of the postulated pathophysiology of MAKI may be similar to those described in cerebral malaria [23]. *Plasmodium falciparum* produces a molecule, Plasmodium falciparum erythrocyte membrane protein 1 (PfEMP1), which is expressed on the surface of infected red cells. This molecule acts as a ligand for the adhesion of the infected red cells to endothelial cells in a process known as sequestration. The resulting endothelial activation leads to expression of more endothelial adhesion molecules and consequently results into compromised renal perfusion [21, 22, 26]. The identification of immunological biomarkers may help to better predict patients at increased risk of MAKI and facilitate earlier interventions [3].

Two- thirds of the AKI patients in the cohort required dialysis. This is similar to findings by Olowu *et al*. where 66% of the children with AKI needed dialysis [9]. In contrast Susantitaphong *et al*. in their meta-analysis of worldwide data found a dialysis need of merely 11%. The two reviews differ in that the former had a population dominated by CA-AKI whereas the latter had children that chiefly had HA-AKI who were predominantly from HIC.

The dialysis access rate for this cohort was 100% even though close to half of the children utilized improvised PD catheters. Olowu *et al* cited reports of similar dialysis access rates from Sudan and South Africa [9]. Similar to Zambia, both these countries have state-funded dialysis programmes. In contrast, the pooled data showed an overall dialysis access rate of only 45% with adults having an access rate of only 49.1%, and children having access of 64% [9]. Given that only patients with overt renal dysfunction are likely to have been identified at referring health facilities it is plausible that the need for dialysis in our population may be higher than reported.

The predictors of the need for dialysis in this cohort were the presence of anuria or oliguria at admission. Due to the small number of patients that had a record of admission serum sodium, potassium, and bicarbonate, these variables were not included in the derivation of the logistic regression model. A multicentre study in Spanish PICU patients found that thrombocytopenia and serum creatinine could predict need for dialysis at discharge from PICU [56]. All the patients in the present cohort had severe AKI thus predictive rules such as the renal angina score would have been unsuitable. More prospective studies that will recruit patients at risk of AKI are needed to better characterise the predictors of severe AKI development and the need for RRT [3].

In children particularly in LICs, the main stay of RRT is PD and this was reflected in our cohort where 88% (82) of all children receiving dialysis underwent this modality. Pooled data from Africa showed a similar PD rate of 80% [9, 16]. Close to half of the catheters used in this cohort, were improvised PD catheters. Others have described the use of improvised PD catheters and fluids [16, 57]. PD is assumed to be cheaper than HD but it is still beyond the reach of many in LICs. Much still needs to be done to secure supplies of child-appropriate dialysis consumables in our population [10, 58].

The overall peritonitis rate was 34.7%. This is lower than rates reported among patients undergoing PD for AKI in two centres in South Africa (47.5% and 41%) but higher than rates reported in DRC [16, 59, 60]. Surprisingly the peritonitis rate was 1.5 higher in association to Tenchoff catheters as compared to improvised catheters (p = 0.07). There is a need to improve infection control measures in the unit.

At discharge from the renal ward, a third of the children had a poor outcome (absconded, CKD, death, ESRD or were discharged for palliation). There was no difference in mortality between patients with MAKI and those without MAKI (5.2% versus 6.1%, p = 0.93). This is similar to findings from a study conducted among Ugandan children, which found that patients with malarial febrile illness and AKI had a mortality of 4.1% when compared to children who had AKI caused by non-malaria febrile illness 4.6% [19]. In contrast, Namazzi *et al*., in another Ugandan study, reported a higher mortality rate of 26.5% among children with stage 3 AKI [18].

The presence of fever, being less than 5 years old and having a higher white cell count were protective from a poor outcome (Table 8). The odds of patients having a poor outcome were increased for children with MAKI but this was not statistically significant (OR 6.4, p = 0.51). This poor sensitivity of various clinical variables to predict MAKI may be because all the patients in the cohort had already developed severe AKI by the time they presented, and predictive models would be more sensitive if applied in populations with early kidney dysfunction.

A year post-presentation with AKI, the mortality rate had risen from 5.6% at the time of discharge to 9.5%. This is lower than the mortality rate of 13.8% that was observed in a meta-analysis of pooled worldwide childhood AKI data [5]. Olowu *et al*. showed an even higher pooled mortality among 1942 children from Africa of 34% [9]. MAKI tends to present as single organ dysfunction in contrast to sepsis-associated AKI, which was the leading aetiology in Olowu *et al*. meta-analysis and may explain the relatively lower mortality reported in our cohort. In addition, mortality rates from the present study may be inaccurate due to the very high rate of loss to follow-up of 77% (97/126 patients), an observation that was also made by Olowu *et al*., [9].

One major factor accounting for this high attrition rate is limited economic resources to permit families to stay out the full course of the treatment. Most patients come from agrarian areas and their families can ill afford to be away from work for extended periods of time. There is need to expand PD facilities to district hospitals which are located in closer proximity to patients especially since recent government policy has been to ensure that at least two medical doctors are available to man such facilities. There is also an urgent requirement to strengthen post-AKI follow-up because children who have suffered severe AKI are at risk of developing CKD [3]. One way this could be done is to empower health personnel at the district level with the necessary skills to monitor and follow up patients with renal dysfunction.

A fifth of children were diagnosed with CKD or ESRD at discharge from the renal ward. This is higher than the pooled rate of 10% reported by Olowu *et al*. [9]. There was no difference in outcome between patients with MAKI and those without AKI caused by other aetiologies (p = 0.35). Namazzi *et al*. reported that 15.6% of Ugandan children still had persistently elevated creatinine a month after suffering from MAKI (Acute kidney disease) [18]. They did not report CKD rates. The CKD rate could have been distorted by the fact that there was no access to the pre-morbid and baseline serum creatinine results of the patients. Thus, pre-existing renal dysfunction could not be determined [9, 11]. In this cohort, 5 children were reported to have reduced cortico-medullary differentiation at admission, but this was not predictive of a poor patient outcome on logistic regression (OR 2.23, p = 0.65). One-year post diagnosis, only four out of the 20 CKD patients were still being followed up. Given this very high loss to follow-up rate, the actual number of children with poor outcomes may, unfortunately, be higher.

This study has several limitations. Firstly, this is a retrospective study therefore not all patient data was available on the folders. Secondly, most of the data on sepsis-associated AKI was not collated because sepsis patients are not followed up by the nephrology service post-PICU and so their folders were unavailable. Thirdly, long-term outcomes for a large proportion of the cohort are unknown because they were lost to follow-up. Also, there is no single test that can specifically diagnose MAKI thus it is possible that MAKI could have been over-estimated. In malaria endemic areas a positive malaria test in the context of AKI may merely represent asymptomatic malaria in a patient with AKI from a different aetiology. Finally, this data was acquired from a paediatric referral centre and may not reflect prevailing situations in other health care situations.

## Conclusions

Over a third of all nephrology patients have AKI and close to three-quarters of AKI patients require dialysis. Malaria is the leading cause of AKI and children from low-medium transmission intensity areas are at risk. There remains a need to improve the ability of staff in peripheral district hospitals to identify and refer AKI early and to institute principles of conservative AKI management before referral.

While all children were able to access dialysis when it was indicated, there was a high catheter use and complication rate, in part due to the high prevalence of utilization of improvised catheters. Yet despite this, the data shows that improvised PD catheters can save lives in contexts where alternatives are not available. Since dialysis is required for a fairly long duration Tenchoff catheter use in AKI patients is advocated for our environment.

A fifth of children developed CKD. Given the high loss to follow-up in this study, these figures could be higher. There is a need for improved follow-up of patients, post-AKI, and this should be done as close to home as possible.

## Supporting information

S1 Appendix Detailed methods used for logistic regression. (DOCX)

S1 Data PLOSONE aetiologies and outcomes of AKI dataset. (XLSX)

S1 File SAS code aetiologies AKI. (DOCX)

S2 File PLOSONE outcome of peritoneal dialysis catheters dataset. (XLSX)
