# Supplementary material for: Malaria is the leading cause of acute kidney injury among a Zambian paediatric renal service cohort retrospectively evaluated for aetiologies, predictors of the need for dialysis, and outcomes
Source: PLoS One. 2023 Oct 25;18(10):e0293037. doi: 10.1371/journal.pone.0293037 (PMC10599569; doi:10.1371/journal.pone.0293037)
Supplement: S1 Appendix — (DOCX) [file pone.0293037.s001.docx]

**Model building process**

**Bi-variable assessments**

The first stage involved bi-variable assessments. Bi-variable assessments of significance of associations between continuous potential predictors and the binary dependent variables were done using the student t-test for difference in means from two independent samples for normally distributed variables, and the Kruskal-Wallis test for difference in distribution of a variable between two independent samples for non-normally distributed variables. Bi-variable assessments of significance of associations between discrete potential predictors and the binary dependent variables were done using chi-square statistics calculated through contingency tables. All contingency tables had two columns only since all the dependent variables had binary responses. Pearson’s chi-square tests were applied on contingency tables with more than 2 rows, and 85% or more cells with expected counts more than 5. The Yate’s- continuity adjusted chi-square test was applied on contingency tables with two rows only, and the Fisher’s exact chi-square test was applied on contingency tables with at least 25% of the cells having expected counts less than 5.

Tested associations that yielded 0.25 or less probability (p)- values were considered statistically significant and included in the second process of determining the predictive models. Variables with known clinical importance were also included in the second stage regardless of their p-value at first stage.

**Multivariable assessments**

The second stage involved building multivariable logistic regression models in a forward-step process. The forward-step process of variable selection is considered objective and can be used when the number of variables under consideration is very large, even larger than the sample size. The method starts with smaller models and is less susceptible to collinearity.[1] The numbers of potential predictors for each of the multivariable assessments in the present study were relatively large and the sample sizes were relatively small, hence the use of the forward-step process of variable selection. The process involved entering potential predictors iteratively based on P-values obtained at bi-variable analysis, beginning with the variable having the lowest P-value, only retaining it in the model if the resulting P-value is 0.25 or less, until the identified potential predictors at bi-variate analysis were all assessed in this manner. Confounding was assessed as a change in any remaining parameter estimate greater than 50% as compared to the full model. Confounders were retained in the models regardless of their p-value.

Statistical significance of each potential predictor in the multivariable models was assessed using the Wald-test. A P-value equal or less than 0.05 was considered indicative of a significant predictor. Confidence intervals were calculated using the Wald method of calculating confidence limits.

The third stage involved adding interaction terms of the retained potential variables in the multivariable models. The only interaction terms to be retained in the multivariable models were those with P-values indicative of a significant predictor.

Assessing quality of the multivariable models was the fourth stage. The models were deemed to be at least of high quality if the model adequately fit the data and one or more of the additional potential predictors were statistically significant. Assessment of at least one of the added predictors being statistically significant was done by applying the likelihood-ratio (LR) chi-square test on the models for the global null hypothesis that none of the added potential predictors predicts the dependent variable. A regression model that fits the data adequately yields predicted values close to the observed values. The models’ ability to fit the data adequately was assessed by applying Hosmer and Lemeshow (H&L) goodness-of-fit test. A logistic regression model that fits the data adequately gives a non-statistically significant p-value in the H&L goodness-of-fit test. [2]

Quality of the model was also judged based on its ability to classify correctly the two responses of the binary outcomes in its predictions. The model’s ability to classify correctly the two responses of the binary outcomes in its predictions was assessed by determining its Concordance ©-statistic. When the C-statistic is greater than 0.7 or greater than 0.8, logistic regression models are usually regarded as reasonable or strong, respectively.[2]

**References**

1. Chowdhury MZI, Turin TC. Variable selection strategies and its importance in clinical prediction modelling. Fam Med Community Health. 2020 Feb 16;8(1):e000262. doi: 10.1136/fmch-2019-000262.

2. Hosmer Jr DW, Lemeshow S, Sturdivant RX. Applied logistic regression. John Wiley & Sons; 2013.
