# Supplementary material for: Malaria is the leading cause of acute kidney injury among a Zambian paediatric renal service cohort retrospectively evaluated for aetiologies, predictors of the need for dialysis, and outcomes
Source: PLoS One. 2023 Oct 25;18(10):e0293037. doi: 10.1371/journal.pone.0293037 (PMC10599569; doi:10.1371/journal.pone.0293037)
Supplement: S1 File — (DOCX) [file pone.0293037.s003.docx]

/***********************************************************

Title: Prevalence aetiology and outcomes

among children at UTH

Purpose: Determining predictor model for MAKI

Author: David Mwakazanga

Co-author Dr Chisambo Mwaba

Date created: November 14, 2022

Date last updated: August 23, 2023

************************************************************/

*created working library;

libname Chisambo 'F:\Working_Folder\Scientists\DrChisambo';

*created formates for variable responses;

**proc** **format** cntlout= Chisambo.aetiolo_newf;

Value $Seasonf **1**= "First Quarter"

**2**= "Second Quarter"

**3**= "Third Quarter"

**4**= "Fourth Quarter";

Value $lusakaprovincepatientf **0**= "No"

**1**= "Yes";

Value $Agecategoryf **1**= "less than 1 year"

**2**= "1 to 5 years"

**3**= "5-10 years"

**4**= "older than 10 years";

Value $Sexf **1**= "Male"

**2**= "Female";

Value $PastmedHxf **1**= "yes"

**2**= "No";

Value $FamHxRenalf **1**= "Yes"

**2**= "No";

Value $Rashf **1**= "Yes"

**2**= "No";

Value $Haemoptysisf **1**= "Yes"

**2**= "No";

Value $coughf **1**= "Yes"

**2**= "No";

Value $odema1f **0**= "No"

**1**= "Yes";

Value $lowgcsf **1**= "Yes"

**2**= "No";

Value $Macroscopichematuriaf **1**= "Yes"

**2**= "No";

Value $oliguriapresentf **1**= "Yes"

**2**= "No";

Value $anuriapresentf **1**= "Yes"

**2**= "No";

Value $arthritisf **1**= "Yes"

**2**= "No";

Value $Seizuresf **1**= "Yes"

**2**= "No";

Value $feverf **1**= "Yes"

**2**= "No";

Value $headachef **1**= "yes"

**2**= "no";

Value $Hypertensionf **1**= "Yes"

**2**= "No";

Value $BPlastfollowupf **1**= "high"

**2**= "normal"

**3**= "low"

**4**= "not done";

Value $ProteinuriaPresentf **1**= "No"

**2**= "1+"

**3**= "2+"

**4**= "3+"

**5**= "4+"

**6**= "Not done";

Value $Haematuriaf **1**= "not done"

**2**= "negative"

**3**= "1+"

**4**= "2+"

**5**= "3+";

Value $RenalOutcomef **1**= ">90ml/1.73/min"

**2**= "GFR 60-89ml/1.73/min"

**3**= "GFR 30-59ml/1.73/min"

**4**= "GFR=15-29ml/1.73/min"

**5**= "GFR < 15 ml/1.73m/min";

Value $finaltreatmentoutcomef **1**= "palliation"

**2**= "dialysis free"

**3**= "home dialysis"

**4**= "death"

**5**= "left against medical advice";

Value $OutcomeOneyearf **1**= "normal renal function"

**2**= "CKD stage 2-4"

**3**= "ESRD on HD"

**4**= "ESRD on PD"

**5**= "ESRD transplanted"

**6**= "lost to followup"

**7**= "Transed out to another facility"

**8**= "Death"

**9**= "followed up less than i year";

Value $OutcomeLastContactf **1**= "Death"

**2**= "Discharged"

**3**= "Absconded"

**4**= "Transferred to another facility"

**5**= "dialysis"

**6**= "transplanted"

**7**= "CKD"

**8**= "normal renal function"

**9**= "palliation";

Value $OUTCOMEf **0**= "Normal kidney function"

**1**= "Death"

**2**= "palliation"

**3**= "Resolving at discharge"

**4**= "CKD"

**5**= "absconded";

Value $OUTCOMEGOODBADf **0**= "Bad"

**1**= "Good";

Value $OUTCOMEBADGOOD1f **0**= "Good"

**1**= "Bad";

Value $kdigo2012stagef **1**= "stage 1"

**2**= "stage 2"

**3**= "stage 3"

**4**= "normal";

Value $peakkidgo2012stagef **1**= "stage 1"

**2**= "stage 2"

**3**= "stage 3";

Value $HIVstausf **1**= "positive"

**2**= "negative"

**3**= "not done";

Value $HepatitisBf **1**= "positive"

**2**= "negative"

**3**= "not done";

Value $HepatitisCf **1**= "positive"

**2**= "negative"

**3**= "not done";

Value $RPRf **1**= "positive"

**2**= "Negative"

**3**= "not done";

Value $RDTmalariaf **1**= "positive"

**2**= "negative"

**3**= "not done";

Value $MPSf **1**= "positive"

**2**= "negative"

**3**= "not done";

Value $KUBUSechogenicf **1**= "normal"

**2**= "reduced"

**3**= "increased"

**4**= "descrepancy in echogenicity"

**5**= "Not done";

Value $KUBUSCMDf **1**= "normal,preserved"

**2**= "Reduced"

**3**= "not reported";

Value $KUBhydronephrosisf **1**= "Yes"

**2**= "No"

**3**= "not done kub";

Value $KUBUSSizef **1**= "Normal"

**2**= "Reduced"

**3**= "enlarged"

**4**= "discrepancy in kidney sizes"

**5**= "Not done";

Value $ANAf **1**= "positive"

**2**= "negative"

**3**= "not done";

Value $C3f **1**= "high"

**2**= "low"

**3**= "normal"

**4**= "not done";

Value $C4f **1**= "high"

**2**= "low"

**3**= "normal"

**4**= "not done";

Value $pANCAf **1**= "high"

**2**= "low"

**3**= "normal"

**4**= "not done";

Value $cANCAf **1**= "high"

**2**= "low"

**3**= "normal"

**4**= "not done";

Value $mechanicalventilationf **1**= "Yes"

**2**= "No";

Value $Aetiologycategoryf **0**= "malaria"

**1**= "glomerulonephritis"

**2**= "HUS"

**3**= "PUV"

**4**= "Sepsis"

**5**= "Hypovolaemia"

**9**= "others";

Value $malariaornotf **0**= "No"

**1**= "Yes";

Value $wasdialysisdonef **0**= "No"

**1**= "Yes";

Value $typeofdialysisdonef **1**= "No"

**2**= "Haemodialysis"

**3**= "Peritoneal Dialysis"

**4**= "both PD and HD";

Value $Aetiologycategory1f **1**= "malaria"

**0**= "Others";/*Dummy variable of Aetiologycategory*/

Value $BPlastfollowup1f **1**= "high"

**0**= "normal";/*Dummy variable of BPlastfollowup*/

Value $Haematuria1f **0**= "No"

**1**= "Yes";/*Dummy variable of Haematuria*/

**run**;

*merged formats into the dataset;

**data** Chisambo.aetiolo_final;

set Chisambo.aetiolo_new;

label

ANA= 'antinuclear antibody'

AetiologyAKI= 'what is the aetiology aki'

Aetiologycategory= 'What category does the aetiology fall into?'

Aetiologycategory1= 'What category does the aetiology fall into?'/*Dummy variable of Aetiologycategory*/

AgePatient= 'Age of patient at presentation'

Agecategory= 'age ategory'

BPlastfollowup= 'was the BP normal at last followup'

BPlastfollowup1= 'was the BP normal at last followup'

C3= 'complement protein 3'

C4= 'complement protein 4'

Creatinine= 'Serum creatinine at admission'

DatePresent= 'Date when subject first presented to hospital with aki'

DrugHX= 'drug history prior to presentation'

DurationPD= 'How many days did patient recieve peritoneal dialysis'

Durationfollup= 'how many moths folllow up'

Durationsymptoms= 'Duration of symptoms prior to presentation'

FamHxRenal= 'Family History of renal disease'

HIVstaus= 'Patient HIV status'

Haematuria= 'Did patient have haematuria at admission'

Haemoglobin= 'Haemoglobin at admission'

Haemoptysis= 'haemoptysispresent'

Height= 'Height of patient'

HepatitisB= 'Patient Hepatitis Bsurface antigen status'

HepatitisC= 'Patient Hepatitis C status'

Hypertension= 'Was patient hypertensive at presentation'

KUBUSCMD= 'KUB US corticomedullary differntiation'

KUBUSCMD2= 'KUB US cortiomedullary differentiation only for those were it was done'

KUBUSSize= 'KUBUS kidney size'

KUBUSechogenic= 'KUB US echogenicity'

KUBhydronephrosis= 'did patienthave hydronephrosis'

MPS= 'Malaria parasite slide'

Macroscopichematuria= 'presentedmacroscpichematuria'

NumberCatheters= 'Number of peritoneal catheters inserted'

OUTCOME= 'consolidated patient outcome'

OUTCOMEBADGOOD1= 'Was patient outcome Bad or Good'

OUTCOMEGOODBAD= 'Was patient outcome good or bad'

OutcomeLastContact= 'Patient Outcome at last contact'

OutcomeOneyear= 'What was thr renal outcome at one year'

PastmedHx= 'significant past medical history'

Potassium= 'Serum potassium at admission'

PresentBPdiastolic= 'Presentation diastolic blood pressure'

PresentBPsystolic= 'presentation systolic BP'

ProteinuriaPresent= 'Did patient have protinuria at presentation'

RDTmalaria= 'Rapid malaria Test Result'

RPR= 'Patient syphyllis(RPR) status'

Rash= 'Rashpresent'

ReasonRef= 'Reason for the Referral'

RefHospital= 'refferring hospital'

RenalOutcome= 'what level of GFR did the patient have at last contact'

Season= 'what quater of the year did patient present'

Seizures= 'seizurespresent'

Sex= 'Sex of Patient'

TimeToPeakCreatinine= 'Days to peak creatinine'

Weight= 'Weight of patient'

Whitecellcount= 'white cell count at admission'

Yearseason= 'What year quater did patient present?'

agecategory2= 'age categorised into 3 intervals for purpose of regression'

anuriapresent= 'presentedanuric?'

arthritis= 'arthritispresent'

cANCA= 'cANCA'

cough= 'coughpresent'

creatininerenaloutcome= 'what was the serum creatinineat last contact'

fever= 'fever at presentation?'

finaltreatmentoutcome= 'what was outcome of treatment'

headache= 'Headacheyesorno'

ifsoPMhx= 'what is past med history'

kdigo2012stage= 'what was the KDIGO2012 stage at presentation'

lengthhospitstay= 'how long did the patient stay in hospital'

lowgcs= 'lowgcs?'

lusakaprovincepatient= 'Is the patient from lusaka province?'

malariaornot= 'Did patient have malaria associated AKI?'

mechanicalventilation= 'was patient mechanically ventilated'

odema1= 'was odema present'

oliguriapresent= 'presentedoliguric?'

otherImaging= 'any other imaging findings'

otherpresentingsymptoms= 'otherpresentingsymptoms'

pANCA= 'pANCA'

peakcreatinine= 'what was the peak creatinine'

peakkidgo2012stage= 'what was the peak kdigo 2012 stage'

platelets= 'platelet count at admission'

sodium= 'Serum sodium at admission'

studynumber= 'subject study number'

typeofdialysisdone= 'what type of dialysis was done'

urea= 'Serum urea at admission'

wasdialysisdone= 'was dialysis conducted?'

whoheightforagemedian= 'what is the WHO median height for age'

;

if Aetiologycategory= '2' then Aetiologycategory1= '0';

if Aetiologycategory= '5' then Aetiologycategory1= '0';

if Aetiologycategory= '3' then Aetiologycategory1= '0';

if Aetiologycategory= '4' then Aetiologycategory1= '0';

if Aetiologycategory= '1' then Aetiologycategory1= '0';

if Aetiologycategory= '0' then Aetiologycategory1= '1';

if BPlastfollowup= '1' then BPlastfollowup1= '1';

if BPlastfollowup= '2' then BPlastfollowup1= '0';

if BPlastfollowup= '3' then BPlastfollowup1= ' ';

if BPlastfollowup= '4' then BPlastfollowup1= ' ';

if BPlastfollowup= ' ' then BPlastfollowup1= ' ';

if lusakaprovincepatient= '.' then lusakaprovincepatient= ' ';

if Hypertension= '.' then Hypertension= ' ';

if Haematuria= '.' then Haematuria1= ' ';

if Haematuria= '1' then Haematuria1= ' ';

if Haematuria= '2' then Haematuria1= '0';

if Haematuria= '3' then Haematuria1= '1';

if Haematuria= '4' then Haematuria1= '1';

if Haematuria= '5' then Haematuria1= '1';

label Haematuria1= 'Did patient have haematuria at admission'

;

format

Aetiologycategory $Aetiologycategoryf.

Agecategory $Agecategoryf.

ANA $ANAf.

anuriapresent $anuriapresentf.

arthritis $arthritisf.

BPlastfollowup $BPlastfollowupf.

C3 $C3f.

C4 $C4f.

cANCA $cANCAf.

cough $coughf.

FamHxRenal $FamHxRenalf.

fever $feverf.

finaltreatmentoutcome $finaltreatmentoutcomef.

Haematuria $Haematuriaf.

Haemoptysis $Haemoptysisf.

headache $headachef.

HepatitisB $HepatitisBf.

HepatitisC $HepatitisCf.

HIVstaus $HIVstausf.

Hypertension $Hypertensionf.

kdigo2012stage $kdigo2012stagef.

KUBhydronephrosis $KUBhydronephrosisf.

KUBUSCMD $KUBUSCMDf.

KUBUSechogenic $KUBUSechogenicf.

KUBUSSize $KUBUSSizef.

lowgcs $lowgcsf.

lusakaprovincepatient $lusakaprovincepatientf.

Macroscopichematuria $Macroscopichematuriaf.

malariaornot $malariaornotf.

mechanicalventilation $mechanicalventilationf.

MPS $MPSf.

odema1 $odema1f.

oliguriapresent $oliguriapresentf.

OUTCOME $OUTCOMEf.

OUTCOMEBADGOOD1 $OUTCOMEBADGOOD1f.

OUTCOMEGOODBAD $OUTCOMEGOODBADf.

OutcomeLastContact $OutcomeLastContactf.

OutcomeOneyear $OutcomeOneyearf.

pANCA $pANCAf.

PastmedHx $PastmedHxf.

peakkidgo2012stage $peakkidgo2012stagef.

ProteinuriaPresent $ProteinuriaPresentf.

Rash $Rashf.

RDTmalaria $RDTmalariaf.

RenalOutcome $RenalOutcomef.

RPR $RPRf.

Season $Seasonf.

Seizures $Seizuresf.

Sex $Sexf.

typeofdialysisdone $typeofdialysisdonef.

wasdialysisdone $wasdialysisdonef.

Aetiologycategory1 $Aetiologycategory1f.

BPlastfollowup1 $BPlastfollowup1f.

Haematuria1 $Haematuria1f.

;

**run**;

*explored database contents;

title1 'Prevalence aetiology and outcomes among children at UTH';

title2 'Database contents';

**proc** **contents** data= Chisambo.aetiolo_final;

**run**;

/***********************************************************

DESCRIPTIVE ANALYSES

************************************************************/

*TABLE 4 Statistics;

*(1) Table 4: Sex;

*Table 4 - Frequency and percentage distributions for Sex and

Sex by malariaornot. Assessed significance of Sex by malariaornot

using continuity adjusted chi-square test;

title2 'Sex, Sex by malariaornot';

**proc** **freq** data= Chisambo.aetiolo_final;

tables Sex Sex*malariaornot/ norow chisq;

**run**;

*not significant at p= 0.1000 (10%)level of significance:

Sex (p= 0.4844).

*(2) Table 4: Age (years)non-categorized;

*Table 4 - AgePatient, AgePatient by wasdialysisdone;

title2 'AgePatient with nomal plot and test for normality';

**proc** **univariate** data= Chisambo.aetiolo_final normal;

var AgePatient;

histogram/ normal;

**run**;

*AgePatient is not normally distributed (Shapiro-Wilk's test p-value= 0.0083).

Use median and interquartile range to describe the data;

*sorted data into the two groups of wasdialysisdone to facilitate

comparison of parameters between the two;

**proc** **sort** data= Chisambo.aetiolo_final;

by malariaornot;

**run**;

*Table 4 - Means for AgePatient by malariaornot;

title2 'Means for AgePatient';

**proc** **means** data= Chisambo.aetiolo_final n nmiss mean std median q1 q3 mode min max;

var AgePatient;

**run**;

title2 'AgePatient by malariaornot';

**proc** **means** data= Chisambo.aetiolo_final n nmiss mean std median q1 q3 mode min max;

var AgePatient;

by malariaornot;

**run**;

*assessed significance of AgePatient by malariaornot using Kruskal Walis;

title2 'Test AgePatient by malariaornot using Kruskal Walis';

**proc** **npar1way** wilcoxon correct= no data= Chisambo.aetiolo_final;

class malariaornot;

var AgePatient;

**run**;

*not significant at p= 0.1000 (10%)level of significance:

AgePatient (p= 0.9121)

*(3) Table 4: Age (years) categorized

*Table 4 - Frequency and percentage distributions for

Agecategory and Agecategory by malariaornot. Assessed

significance of Agecategory by wasdialysisdon using fisher's exact test;

title2 'Agecategory, Agecategory by malariaornot';

**proc** **freq** data= Chisambo.aetiolo_final;

tables Agecategory Agecategory*malariaornot/ norow chisq;

exact fisher;

**run**;

*significant at p= 0.1000 (10%)level of significance:

Agecategory (p= 0.0249)

*(4) Table 4: Referring Province;

*Table 4 - Frequency and percentage distributions for

lusakaprovincepatient and lusakaprovincepatient by malariaornot.

Assessed significance of Sex by malariaornot using continuity adjusted chi-square test;

title2 'lusakaprovincepatient, lusakaprovincepatient by malariaornot';

**proc** **freq** data= Chisambo.aetiolo_final;

tables lusakaprovincepatient lusakaprovincepatient*malariaornot/ norow chisq;

exact fisher;

**run**;

*not significant at p= 0.1000 (10%)level of significance:

lusakaprovincepatient (p= 0.0868)

*(5) Table 4: Height;

*Table 4 - Height, Height by malariaornot;

title2 'Height with nomal plot and test for normality';

**proc** **univariate** data= Chisambo.aetiolo_final normal;

var Height;

histogram/ normal;

**run**;

*Height is normally distributed (Shapiro-Wilk's test p-value= 0.0648).

Use mean and standard deviation to describe the data;

*sorted data into the two groups of malariaornot to facilitate

comparison of parameters between the two;

**proc** **sort** data= Chisambo.aetiolo_final;

by malariaornot;

**run**;

*Table 4 - Means for Height by malariaornot;

title2 'Height by malariaornot';

**proc** **means** data= Chisambo.aetiolo_final n nmiss mean std median q1 q3 mode min max;

var Height;

by malariaornot;

**run**;

*assessed significance of AgePatient by malariaornot using pooled ttest test,

the variances are not equal (p-value= 0.5558;

title2 'Test Height by malariaornot using ttest';

**proc** **ttest** data= Chisambo.aetiolo_final;

class malariaornot;

var Height;

**run**;

*not significant at p= 0.1000 (10%)level of significance:

Height (p= 0.3351).

*(6) Table 4: Weight;

*Table 4 - Weight, Weight by malariaornot;

title2 'Weight with nomal plot and test for normality';

**proc** **univariate** data= Chisambo.aetiolo_final normal;

var Weight;

histogram/ normal;

**run**;

*Weight is not normally distributed (Shapiro-Wilk's test p-value< 0.0001).

Use median and interquartile range to describe the data;

*sorted data into the two groups of malariaornot to facilitate

comparison of parameters between the two;

**proc** **sort** data= Chisambo.aetiolo_final;

by malariaornot;

**run**;

*Table 4 - Means for Weight by malariaornot;

title2 'Means for Weight';

**proc** **means** data= Chisambo.aetiolo_final n nmiss mean std median q1 q3 mode min max;

var Weight;

**run**;

title2 'Weight by malariaornot';

**proc** **means** data= Chisambo.aetiolo_final n nmiss mean std median q1 q3 mode min max;

var Weight;

by malariaornot;

**run**;

*assessed significance of Weight by malariaornot using Kruskal Walis;

title2 'Test Weight by malariaornot using Kruskal Walis';

**proc** **npar1way** wilcoxon correct= no data= Chisambo.aetiolo_final;

class malariaornot;

var Weight;

**run**;

*not significant at p= 0.1000 (10%)level of significance:

Weight (p= 0.9981)

*(7) Table 4: Illness Duration;

*Table 4 - Durationsymptoms, Durationsymptoms by malariaornot;

title2 'Durationsymptoms with nomal plot and test for normality';

**proc** **univariate** data= Chisambo.aetiolo_final normal;

var Durationsymptoms;

histogram/ normal;

**run**;

*Durationsymptoms is not normally distributed (Shapiro-Wilk's test p-value< 0.0001).

Use median and interquartile range to describe the data;

*sorted data into the two groups of malariaornot to facilitate

comparison of parameters between the two;

**proc** **sort** data= Chisambo.aetiolo_final;

by malariaornot;

**run**;

*Table 4 - Means for Durationsymptoms by malariaornot;

title2 'Means for Durationsymptoms';

**proc** **means** data= Chisambo.aetiolo_final n nmiss mean std median q1 q3 mode min max;

var Durationsymptoms;

**run**;

title2 'Durationsymptoms by malariaornot';

**proc** **means** data= Chisambo.aetiolo_final n nmiss mean std median q1 q3 mode min max;

var Durationsymptoms;

by malariaornot;

**run**;

*assessed significance of Durationsymptoms by malariaornot using Kruskal Walis;

title2 'Test Durationsymptoms by malariaornot using Kruskal Walis';

**proc** **npar1way** wilcoxon correct= no data= Chisambo.aetiolo_final;

class malariaornot;

var Durationsymptoms;

**run**;

*not significant at p= 0.1000 (10%)level of significance:

Durationsymptoms (p= 0.0427);

*(8) Table 4: Season;

*Table 4 - Frequency and percentage distributions for

Season and Season by malariaornot. Assessed

significance of Season by wasdialysisdon using Chi-square test;

title2 'Season, Season by malariaornot';

**proc** **freq** data= Chisambo.aetiolo_final;

tables Season Season*malariaornot/ norow chisq;

exact fisher;

**run**;

*not significant at p= 0.1000 (10%)level of significance:

Season (p= 0.0058)

*(9) Table 4: Oedema;

*Table 4 - Frequency and percentage distributions for

Oedema1 and Oedema1 by malariaornot. Assessed

significance of Oedema1 by wasdialysisdon using continuity

adjusted Chi-square test;

title2 'Odema1, Odema1 by malariaornot';

**proc** **freq** data= Chisambo.aetiolo_final;

tables Odema1 Odema1*malariaornot/ norow chisq;

exact fisher;

**run**;

*not significant at p= 0.1000 (10%)level of significance:

Oedema1 (p= 0.6376)

*(10) Table 4: Gross Haematuria

*Table 4 - Frequency and percentage distributions for

Haematuria1 and Haematuria1 by malariaornot. Assessed

significance of Haematuria1 by wasdialysisdon using continuity

adjusted Chi-square test;

title2 'Haematuria1, Haematuria1 by malariaornot';

**proc** **freq** data= Chisambo.aetiolo_final;

tables Haematuria1 Haematuria1*malariaornot/ norow chisq;

exact fisher;

**run**;

*not significant at p= 0.1000 (10%)level of significance:

Haematuria1 (p= 0.8042)

*(11) Table 4: Anuria

*Table 4 - Frequency and percentage distributions for

anuriapresent and anuriapresent by malariaornot. Assessed

significance of anuriapresent by wasdialysisdon using continuity

adjusted Chi-square test;

title2 'anuriapresent, anuriapresent by malariaornot';

**proc** **freq** data= Chisambo.aetiolo_final;

tables anuriapresent anuriapresent*malariaornot/ norow chisq;

exact fisher;

**run**;

*significant at p= 0.1000 (10%)level of significance:

anuriapresent (p= 1.0000)

*(12) Table 4: Oliguria

*Table 4 - Frequency and percentage distributions for

oliguriapresent and oliguriapresent by malariaornot. Assessed

significance of oliguriapresent by wasdialysisdon using continuity

adjusted Chi-square test;

title2 'oliguriapresent, oliguriapresent by malariaornot';

**proc** **freq** data= Chisambo.aetiolo_final;

tables oliguriapresent oliguriapresent*malariaornot/ norow chisq;

exact fisher;

**run**;

*not significant at p= 0.1000 (10%)level of significance:

oliguriapresent (p= 0.6606)

(13) Table 5: Reduced GCS;

*Table 5 - Frequency and percentage distributions for

lowgcs and lowgcs by malariaornot. Assessed

significance of lowgcs by wasdialysisdon using Fisher's

exact test;

title2 'lowgcs, lowgcs by malariaornot';

**proc** **freq** data= Chisambo.aetiolo_final;

tables lowgcs lowgcs*malariaornot/ norow chisq;

exact fisher;

**run**;

*not significant at p= 0.1000 (10%)level of significance:

lowgcs (p= 0.3554)

*(14) Table 4: Fever

*Table 4 - Frequency and percentage distributions for

fever and fever by malariaornot. Assessed

significance of fever by wasdialysisdon using continuity

adjusted Chi-square test;

title2 'fever, fever by malariaornot';

**proc** **freq** data= Chisambo.aetiolo_final;

tables fever fever*malariaornot/ norow chisq;

exact fisher;

**run**;

*not significant at p= 0.1000 (10%)level of significance:

fever (p= 0.0006)

*(15) Table 4: Hypertension;

*Table 4 - Frequency and percentage distributions for

Hypertension and Hypertension by malariaornot. Assessed

significance of Hypertension by wasdialysisdon using continuity

adjusted Chi-square test;

title2 'Hypertension, Hypertension by malariaornot';

**proc** **freq** data= Chisambo.aetiolo_final;

tables Hypertension Hypertension*malariaornot/ norow chisq;

exact fisher;

**run**;

*significant at p= 0.1000 (10%)level of significance:

Hypertension (p= <.0001)

*(16)Table 4: Admission Haemoglobin;

*Table 4 - Haemoglobin, Haemoglobin by malariaornot;

title2 'Haemoglobin with nomal plot and test for normality';

**proc** **univariate** data= Chisambo.aetiolo_final normal;

var Haemoglobin;

histogram/ normal;

**run**;

*Haemoglobin is not normally distributed (Shapiro-Wilk's test p-value< 0.0139).

Use median and interquartile range to describe the data;

*sorted data into the two groups of malariaornot to facilitate

comparison of parameters between the two;

**proc** **sort** data= Chisambo.aetiolo_final;

by malariaornot;

**run**;

*Table 4 - Means for Haemoglobin by malariaornot;

title2 'Means for Haemoglobin';

**proc** **means** data= Chisambo.aetiolo_final n nmiss mean std median q1 q3 mode min max;

var Haemoglobin;

**run**;

title2 'Haemoglobin by malariaornot';

**proc** **means** data= Chisambo.aetiolo_final n nmiss mean std median q1 q3 mode min max;

var Haemoglobin;

by malariaornot;

**run**;

*assessed significance of Haemoglobin by malariaornot using Kruskal Walis;

title2 'Test Haemoglobin by malariaornot using Kruskal Walis';

**proc** **npar1way** wilcoxon correct= no data= Chisambo.aetiolo_final;

class malariaornot;

var Haemoglobin;

**run**;

*significant at p= 0.1000 (10%)level of significance:

Haemoglobin (p= 0.0237)

*(17) Table 4: White cell count;

*Table 4 - Whitecellcount, Whitecellcount by malariaornot;

title2 'Whitecellcount with nomal plot and test for normality';

**proc** **univariate** data= Chisambo.aetiolo_final normal;

var Whitecellcount;

histogram/ normal;

**run**;

*Whitecellcount is not normally distributed (Shapiro-Wilk's test p-value< 0.0001).

Use median and interquartile range to describe the data;

*sorted data into the two groups of malariaornot to facilitate

comparison of parameters between the two;

**proc** **sort** data= Chisambo.aetiolo_final;

by malariaornot;

**run**;

*Table 4 - Means for Whitecellcount by malariaornot;

title2 'Means for Whitecellcount';

**proc** **means** data= Chisambo.aetiolo_final n nmiss mean std median q1 q3 mode min max;

var Whitecellcount;

**run**;

title2 'Whitecellcount by malariaornot';

**proc** **means** data= Chisambo.aetiolo_final n nmiss mean std median q1 q3 mode min max;

var Whitecellcount;

by malariaornot;

**run**;

*assessed significance of Whitecellcount by malariaornot using Kruskal Walis;

title2 'Test Whitecellcount by malariaornot using Kruskal Walis';

**proc** **npar1way** wilcoxon correct= no data= Chisambo.aetiolo_final;

class malariaornot;

var Whitecellcount;

**run**;

*not significant at p= 0.1000 (10%)level of significance:

Whitecellcount (p= 0.2921)

*(18) Table 4: Admission platelet count;

*Table 4 - platelets, platelets by wasdialysisdone;

title2 'platelets with nomal plot and test for normality';

**proc** **univariate** data= Chisambo.aetiolo_final normal;

var platelets;

histogram/ normal;

**run**;

*platelets is not normally distributed (Shapiro-Wilk's test p-value< 0.0001).

Use median and interquartile range to describe the data;

*sorted data into the two groups of wasdialysisdone to facilitate

comparison of parameters between the two;

**proc** **sort** data= Chisambo.aetiolo_final;

by malariaornot;

**run**;

*Table 4 - Means for platelets by malariaornot;

title2 'Means for platelets';

**proc** **means** data= Chisambo.aetiolo_final n nmiss mean std median q1 q3 mode min max;

var platelets;

**run**;

title2 'platelets by malariaornot';

**proc** **means** data= Chisambo.aetiolo_final n nmiss mean std median q1 q3 mode min max;

var platelets;

by malariaornot;

**run**;

*assessed significance of platelets by wasdialysisdone using Kruskal Walis;

*not significant at p= 0.1000 (10%)level of significance:

platelets (p= 0.1472)

*(19) Table 4: Admission serum soldium;

*Table 1 - sodium, sodium by malariaornot;

title2 'sodium with nomal plot and test for normality';

**proc** **univariate** data= Chisambo.aetiolo_final normal;

var sodium;

histogram/ normal;

**run**;

*sodium is not normally distributed (Shapiro-Wilk's test p-value< 0.0001).

Use median and interquartile range to describe the data;

*sorted data into the two groups of malariaornot to facilitate

comparison of parameters between the two;

**proc** **sort** data= Chisambo.aetiolo_final;

by malariaornot;

**run**;

*Table 4 - Means for sodium by malariaornot;

title2 'Means for sodium';

**proc** **means** data= Chisambo.aetiolo_final n nmiss mean std median q1 q3 mode min max;

var sodium;

**run**;

title2 'sodium by malariaornot';

**proc** **means** data= Chisambo.aetiolo_final n nmiss mean std median q1 q3 mode min max;

var sodium;

by malariaornot;

**run**;

*assessed significance of sodium by malariaornot using Kruskal Walis;

title2 'Test sodium by malariaornot using Kruskal Walis';

**proc** **npar1way** wilcoxon correct= no data= Chisambo.aetiolo_final;

class malariaornot;

var sodium;

**run**;

*significant at p= 0.1000 (10%)level of significance:

sodium (p= 0.0204)

*(20) Table 4: Admission serum creatinine;

*Table 4 - creatinine, creatinine by malariaornot;

title2 'creatinine with nomal plot and test for normality';

**proc** **univariate** data= Chisambo.aetiolo_final normal;

var creatinine;

histogram/ normal;

**run**;

*creatinine is not normally distributed (Shapiro-Wilk's test p-value< 0.0010).

Use mean and interquartile range to describe the data;

*sorted data into the two groups of malariaornot to facilitate

comparison of parameters between the two;

**proc** **sort** data= Chisambo.aetiolo_final;

by malariaornot;

**run**;

*Table 4 - Means for creatinine by malariaornot;

title2 'Means for creatinine';

**proc** **means** data= Chisambo.aetiolo_final n nmiss mean std median q1 q3 mode min max;

var creatinine;

**run**;

title2 'creatinine by malariaornot';

**proc** **means** data= Chisambo.aetiolo_final n nmiss mean std median q1 q3 mode min max;

var creatinine;

by malariaornot;

**run**;

*assessed significance of creatinine by malariaornot using Kruskal Walis;

title2 'Test creatinine by malariaornot using Kruskal Walis';

**proc** **npar1way** wilcoxon correct= no data= Chisambo.aetiolo_final;

class malariaornot;

var creatinine;

**run**;

*significant at p= 0.1000 (10%)level of significance:

creatinine (p= 0.0537)

*(21) Table 4: Days to peak creatinine

*Table 4 - TimeToPeakCreatinine, TimeToPeakCreatinine by malariaornot;

title2 'TimeToPeakCreatinine with nomal plot and test for normality';

**proc** **univariate** data= Chisambo.aetiolo_final normal;

var TimeToPeakCreatinine;

histogram/ normal;

**run**;

*TimeToPeakCreatinine is not normally distributed (Shapiro-Wilk's test p-value< 0.0001).

Use median and interquartile range to describe the data;

*sorted data into the two groups of malariaornot to facilitate

comparison of parameters between the two;

**proc** **sort** data= Chisambo.aetiolo_final;

by malariaornot;

**run**;

*Table 4 - Means for TimeToPeakCreatinine by malariaornot;

title2 'Means for TimeToPeakCreatinine';

**proc** **means** data= Chisambo.aetiolo_final n nmiss mean std median q1 q3 mode min max;

var TimeToPeakCreatinine;

**run**;

title2 'TimeToPeakCreatinine by malariaornot';

**proc** **means** data= Chisambo.aetiolo_final n nmiss mean std median q1 q3 mode min max;

var TimeToPeakCreatinine;

by malariaornot;

**run**;

*assessed significance of TimeToPeakCreatinine by malariaornot using Kruskal Walis;

title2 'Test TimeToPeakCreatinine by malariaornot using Kruskal Walis';

**proc** **npar1way** wilcoxon correct= no data= Chisambo.aetiolo_final;

class malariaornot;

var TimeToPeakCreatinine;

**run**;

*not significant at p= 0.1000 (10%)level of significance:

TimeToPeakCreatinine (p <0.2053)

*(22) Table 4: Peak creatinine;

*Table 4 - peakcreatinine, peakcreatinine by malariaornot;

title2 'peakcreatinine with nomal plot and test for normality';

**proc** **univariate** data= Chisambo.aetiolo_final normal;

var peakcreatinine;

histogram/ normal;

**run**;

*peakcreatinine is not normally distributed (Shapiro-Wilk's test p-value< 0.042).

Use median and interquartile range to describe the data;

*sorted data into the two groups of malariaornot to facilitate

comparison of parameters between the two;

**proc** **sort** data= Chisambo.aetiolo_final;

by malariaornot;

**run**;

*Table 4 - Means for peakcreatinine by malariaornot;

title2 'Means for peakcreatinine';

**proc** **means** data= Chisambo.aetiolo_final n nmiss mean std median q1 q3 mode min max;

var peakcreatinine;

**run**;

title2 'peakcreatinine by malariaornot';

**proc** **means** data= Chisambo.aetiolo_final n nmiss mean std median q1 q3 mode min max;

var peakcreatinine;

by malariaornot;

**run**;

*assessed significance of peakcreatinine by malariaornot using Kruskal Walis;

title2 'Test peakcreatinine by malariaornot using Kruskal Walis';

**proc** **npar1way** wilcoxon correct= no data= Chisambo.aetiolo_final;

class malariaornot;

var peakcreatinine;

**run**;

*significant at p= 0.1000 (10%)level of significance:

peakcreatinine (p= 0.0065)

*(23) Table 4: KDIGO AKI stage

*Table 4 - Frequency and percentage distributions for

peakkidgo2012stage and peakkidgo2012stage by malariaornot. Assessed

significance of peakkidgo2012stage by wasdialysisdon using Fisher's

exact test;

title2 'peakkidgo2012stage, peakkidgo2012stage by malariaornot';

**proc** **freq** data= Chisambo.aetiolo_final;

tables peakkidgo2012stage peakkidgo2012stage*malariaornot/ norow chisq;

exact fisher;

**run**;

*not significant at p= 0.1000 (10%)level of significance:

peakkidgo2012stage (p= 1.0000)

*(24) Table 4: HIV;

*Table 4 - Frequency and percentage distributions for

HIVstaus and HIVstaus by malariaornot. Assessed

significance of HIVstaus by wasdialysisdon using Fisher's

exact test;

title2 'HIVstaus, HIVstaus by malariaornot';

**proc** **freq** data= Chisambo.aetiolo_final;

tables HIVstaus HIVstaus*malariaornot/ norow chisq;

exact fisher;

**run**;

*not significant at p= 0.1000 (10%)level of significance:

HIVstaus (p= 0.3554)

*(25) Table 4: Dialyzed

*Table 4 - Frequency and percentage distributions for

wasdialysisdone and wasdialysisdone by malariaornot. Assessed

significance of wasdialysisdone by malariaornot using

continuity adjusted chisquare test;

title2 'wasdialysisdone, wasdialysisdone by malariaornot';

**proc** **freq** data= Chisambo.aetiolo_final;

tables wasdialysisdone wasdialysisdone*malariaornot/ norow chisq;

exact fisher;

**run**;

*not significant at p= 0.1000 (10%)level of significance:

wasdialysisdone (p= 0.3881)

*(26) Table 4: Illness Duration;

*Table 4 - DurationPD, DurationPD by malariaornot;

title2 'DurationPD with nomal plot and test for normality';

**proc** **univariate** data= Chisambo.aetiolo_final normal;

var DurationPD;

histogram/ normal;

**run**;

*DurationPD is not normally distributed (Shapiro-Wilk's test p-value< 0.0001).

Use median and interquartile range to describe the data;

*sorted data into the two groups of malariaornot to facilitate

comparison of parameters between the two;

**proc** **sort** data= Chisambo.aetiolo_final;

by malariaornot;

**run**;

*Table 4 - Means for DurationPD by malariaornot;

title2 'Means for DurationPD';

**proc** **means** data= Chisambo.aetiolo_final n nmiss mean std median q1 q3 mode min max;

var DurationPD;

**run**;

title2 'DurationPD by malariaornot';

**proc** **means** data= Chisambo.aetiolo_final n nmiss mean std median q1 q3 mode min max;

var DurationPD;

by malariaornot;

**run**;

*assessed significance of DurationPD by malariaornot using Kruskal Walis;

title2 'Test DurationPD by malariaornot using Kruskal Walis';

**proc** **npar1way** wilcoxon correct= no data= Chisambo.aetiolo_final;

class malariaornot;

var DurationPD;

**run**;

*significant at p= 0.1000 (10%)level of significance:

DurationPD (p= 0.0498);

*(27) Table 4: Outcome

*Table 4 - Frequency and percentage distributions for

OUTCOMEGOODBAD and OUTCOMEGOODBAD by wasdialysisdone. Assessed

significance of OUTCOMEGOODBAD by wasdialysisdon using

continuity adjusted chisquare test;

title2 'OUTCOMEGOODBAD, OUTCOMEGOODBAD by malariaornot';

**proc** **freq** data= Chisambo.aetiolo_final;

tables OUTCOMEGOODBAD OUTCOMEGOODBAD*malariaornot/ norow chisq;

exact fisher;

**run**;

*significant at p= 0.1000 (10%)level of significance:

OUTCOMEGOODBAD (p= 0.0062)

*(28) Table 4: Patient outcome Subcategory

*Table 4 - Frequency and percentage distributions for

finaltreatmentoutcome and finaltreatmentoutcome by wasdialysisdone. Assessed

significance of finaltreatmentoutcome by wasdialysisdon using

fisher's exact test;

title2 'finaltreatmentoutcome, finaltreatmentoutcome by malariaornot';

**proc** **freq** data= Chisambo.aetiolo_final;

tables finaltreatmentoutcome finaltreatmentoutcome*malariaornot/ norow chisq;

exact fisher;

**run**;

*not significant at p= 0.1000 (10%)level of significance:

finaltreatmentoutcome (p= 0.1128)

*(29) Table 4: Length hospital stay (days);

*Table 4 - lengthhospitstay, lengthhospitstay by malariaornot;

title2 'lengthhospitstay with nomal plot and test for normality';

**proc** **univariate** data= Chisambo.aetiolo_final normal;

var lengthhospitstay;

histogram/ normal;

**run**;

*lengthhospitstay is not normally distributed (Shapiro-Wilk's test p-value< 0.0001).

Use median and interquartile range to describe the data;

*sorted data into the two groups of malariaornot to facilitate

comparison of parameters between the two;

**proc** **sort** data= Chisambo.aetiolo_final;

by malariaornot;

**run**;

*Table 4 - Means for lengthhospitstay by malariaornot;

title2 'Means for lengthhospitstay';

**proc** **means** data= Chisambo.aetiolo_final n nmiss mean std median q1 q3 mode min max;

var lengthhospitstay;

**run**;

title2 'lengthhospitstay by malariaornot';

**proc** **means** data= Chisambo.aetiolo_final n nmiss mean std median q1 q3 mode min max;

var lengthhospitstay;

by malariaornot;

**run**;

*assessed significance of Durationsymptoms by malariaornot using Kruskal Walis;

title2 'Test lengthhospitstay by malariaornot using Kruskal Walis';

**proc** **npar1way** wilcoxon correct= no data= Chisambo.aetiolo_final;

class malariaornot;

var lengthhospitstay;

**run**;

*significant at p= 0.1000 (10%)level of significance:

lengthhospitstay (p= 0.4943)

*(30) Table 4: Duration follow-up(days);

*Table 4 - Durationfollup, Durationfollup by malariaornot;

title2 'Durationfollup with nomal plot and test for normality';

**proc** **univariate** data= Chisambo.aetiolo_final normal;

var Durationfollup;

histogram/ normal;

**run**;

*Durationfollup is not normally distributed (Shapiro-Wilk's test p-value< 0.0010).

Use median and interquartile range to describe the data;

*sorted data into the two groups of malariaornot to facilitate

comparison of parameters between the two;

**proc** **sort** data= Chisambo.aetiolo_final;

by malariaornot;

**run**;

*Table 4 - Means for Durationfollup by malariaornot;

title2 'Means for Durationfollup';

**proc** **means** data= Chisambo.aetiolo_final n nmiss mean std median q1 q3 mode min max;

var Durationfollup;

**run**;

title2 'Durationfollup by malariaornot';

**proc** **means** data= Chisambo.aetiolo_final n nmiss mean std median q1 q3 mode min max;

var Durationfollup;

by malariaornot;

**run**;

*assessed significance of Durationsymptoms by malariaornot using Kruskal Walis;

title2 'Test Durationfollup by malariaornot using Kruskal Walis';

**proc** **npar1way** wilcoxon correct= no data= Chisambo.aetiolo_final;

class malariaornot;

var Durationfollup;

**run**;

*significant at p= 0.1000 (10%)level of significance:

Durationfollup (p= 0.0951)

/***************************************************

MODEL BUILDING

*included all the variables that were significant at 10% level of

significance and are scientifically plausible, in a forward step

procedure begining with the variable that had the smallest

p-value. Variables will be kept in the model building process if

they continue being significant at 10% level of significance.

lusakaprovincepatient (p= 0.0868)

Durationsymptoms (p= 0.0427)

Season (p= 0.0058)

fever (p= 0.0006)

Hypertension (p= <.0001)

Haemoglobin (p= 0.0237)

sodium (p= 0.0204)

creatinine (p= 0.0537)

peakcreatinine (p= 0.0065)

DurationPD (p= 0.0498)

OUTCOMEGOODBAD (p= 0.0062)

lengthhospitstay (p= 0.0498)

Durationfollup (p= 0.0951)

****************************************************;

*step 1: added Hypertension (p= <.0001);

title2 'Logistic model 1 (Step 1 added Hypertension (p= <.0001))';

**proc** **logistic** data= Chisambo.aetiolo_final;

class Hypertension (ref="Yes");

model malariaornot (event= 'Yes')= Hypertension;

**run**;

*c= 0.699;

*kept: Hypertension (p= <.0001);

*step 2: added fever (p= 0.0006);

title2 'Logistic model 2 (Step 2 added fever (p= 0.0006))';

**proc** **logistic** data= Chisambo.aetiolo_final;

class Hypertension (ref="Yes") fever (ref="Yes");

model malariaornot (event= 'Yes')= Hypertension fever;

**run**;

*c= 0.766;

*kept: Hypertension (p= <.0001), fever (p= 0.0035);

*step 3: added Season (p= 0.006);

title2 'Logistic model 3 (Step 3 added OUTCOMEGOODBAD (p= 0.0062))';

**proc** **logistic** data= Chisambo.aetiolo_final;

class Hypertension fever Season;

model malariaornot (event= 'Yes')= Hypertension fever Season;

**run**;

*c= 0.815;

*kept: Hypertension (p= 0.0008), fever (p= 0.0023), Season (p= 0.0607);

*step 4: added peakcreatinine (p= 0.0065);

title2 'Logistic model 4 (Step 4 added peakcreatinine (p= 0.0065))';

**proc** **logistic** data= Chisambo.aetiolo_final;

class Hypertension fever Season;

model malariaornot (event= 'Yes')= Hypertension fever Season peakcreatinine;

**run**;

*c= 0.851;

*kept: Hypertension (p= 0.0008), fever (p= 0.0013), Season= 0.0667,

peakcreatinine (p= 0.0069);

*step 5: added sodium (p= 0.0204);

title2 'Logistic model 5 (Step 5 added sodium (p= 0.0204))';

**proc** **logistic** data= Chisambo.aetiolo_final;

class Hypertension fever season ;

model malariaornot (event= 'Yes')= Hypertension fever peakcreatinine season sodium;

**run**;

*c= 0.864;

*kept: Hypertension (p= 0.0029), fever (p= 0.0069) peakcreatinine (p= 0.0568)

Sodium (p= 0.0682);

*removed: Season (p= 0.2941);

*step 6: added Haemoglobin (p= 0.0237);

title2 'Logistic model 6 (Step 6 added Haemoglobin (p= 0.0237)';

**proc** **logistic** data= Chisambo.aetiolo_final;

class Hypertension fever;

model malariaornot (event= 'Yes')= Hypertension fever peakcreatinine sodium

Haemoglobin ;

**run**;

*c= 0.865;

*kept: Hypertension (p= 0.0051), fever (p= 0.0215) peakcreatinine (p= 0.0692)

Sodium (p= 0.0263) Haemoglobin (p= 0.0659);

*step 7: Durationsymptoms (p= 0.0427);

title2 'Logistic model 7 (Step 7 added Durationsymptoms (p= 0.0427)';

**proc** **logistic** data= Chisambo.aetiolo_final;

class Hypertension fever;

model malariaornot (event= 'Yes')= Hypertension fever peakcreatinine sodium

Haemoglobin Durationsymptoms;

**run**;

*kept: Hypertension (p= 0.0092), fever (p= 0.1365) peakcreatinine (p= 0.0425)

Sodium (p= 0.0689) Haemoglobin (p= 0.0515);

*Removed: Durationsymptoms (p= 0.2481);

*step 8: creatinine (p= 0.0537);

title2 'Logistic model 9 (Step 9 added creatinine (p= 0.0537)';

**proc** **logistic** data= Chisambo.aetiolo_final;

class Hypertension fever;

model malariaornot (event= 'Yes')= Hypertension fever peakcreatinine sodium

Haemoglobin creatinine;

**run**;

*kept: Hypertension (p= 0.0055), fever (p= 0.0285) peakcreatinine (p= 0.2067)

Sodium (p= 0.0248) Haemoglobin (p= 0.0614);

*removed: creatinine (p= 0.7152);

*step 9: added lusakaprovincepatient (p= 0.0868);

title2 'Logistic model 11 (Step 11 added lusakaprovincepatient (p= 0.0868)';

**proc** **logistic** data= Chisambo.aetiolo_final;

class Hypertension fever lusakaprovincepatient;

model malariaornot (event= 'Yes')= Hypertension fever peakcreatinine sodium

Haemoglobin lusakaprovincepatient;

**run**;

*kept: Hypertension (p= 0.0059), fever (p= 0.0199) peakcreatinine (p= 0.0624)

Sodium (p= 0.0387) Haemoglobin (p= 0.0813);

*removed: lusakaprovincepatient (p= 0.9914);

*step 10: added Durationfollup (p= 0.0951);

title2 'Logistic model 12 (Step 12 added Durationfollup (p= 0.0951)';

**proc** **logistic** data= Chisambo.aetiolo_final;

class Hypertension fever;

model malariaornot (event= 'Yes')= Hypertension fever peakcreatinine sodium

Haemoglobin DurationPD Durationfollup;

;

**run**;

*kept: Hypertension (p= 0.0040), fever (p= 0.0134) peakcreatinine (p= 0.2997)

Sodium (p= 0.0395) Haemoglobin (p= 0.1105) DurationPD (p= 0.0736);

*removed: Durationfollup (p= 0.3012);

*step 11: removed Durationfollup (p= 0.3012);

title2 'Logistic model 12 (Step 12 removed DurationPD (p= 0.0868)';

**proc** **logistic** data= Chisambo.aetiolo_final;

class Hypertension fever;

model malariaornot (event= 'Yes')= Hypertension peakcreatinine fever sodium

Haemoglobin;

;

**run**;

*kept: Hypertension (p= 0.0075), fever (p= 0.0260)Sodium (p= 0.0116)

Haemoglobin (p= 0.0468);

*step 12: added interaction term: Hypertension*Sodium;

title2 'Logistic model 13 (Step 13 added Hypertension*Sodium)';

**proc** **logistic** data= Chisambo.aetiolo_final;

class Hypertension fever;

model malariaornot (event= 'Yes')= Hypertension sodium fever

Haemoglobin Hypertension*Sodium;

;

**run**;

*kept: Hypertension (p= 0.3376), fever (p= 0.0242), Sodium (p= 0.0304)

Haemoglobin (p= 0.0601);

*removed: Hypertension*Sodium (p= 0.4133);

*step 14: added interaction term: Hypertension*fever;

title2 'Logistic model 14 (Step 14 added Hypertension*fever)';

**proc** **logistic** data= Chisambo.aetiolo_final;

class Hypertension fever;

model malariaornot (event= 'Yes')= Hypertension sodium fever

Haemoglobin Hypertension*fever;

;

**run**;

*kept: Hypertension (p= 0.0066), fever (p= 0.0578), Sodium (p= 0.0111)

Haemoglobin (p= 0.0471);

*removed: Hypertension*fever (p= 0.6022);

*step 15: added interaction term: Hypertension*Haemoglobin;

title2 'Logistic model 15 (Step 15 added Hypertension*Haemoglobin)';

**proc** **logistic** data= Chisambo.aetiolo_final;

class Hypertension fever;

model malariaornot (event= 'Yes')= Hypertension sodium fever

Haemoglobin Hypertension*Haemoglobin;

;

**run**;

*kept: Hypertension (p= 0.3949), fever (p= 0.0084), Sodium (p= 0.0241)

Haemoglobin (p= 0.0465);

*removed: Hypertension*fever (p= 0.1617);

*step 16: added interaction term: Hypertension*Haemoglobin;

title2 'Logistic model 16 (Step 16 added Hypertension*Haemoglobin) (p= 0.0868)';

**proc** **logistic** data= Chisambo.aetiolo_final;

class Hypertension fever;

model malariaornot (event= 'Yes')= Hypertension sodium fever

Haemoglobin Hypertension*Haemoglobin;

;

**run**;

*kept: Hypertension (p= 0.3949), fever (p= 0.0084), Sodium (p= 0.0241)

Haemoglobin (p= 0.0465);

*removed: Hypertension*Haemoglobin (p= 0.1617);

*step 17: added interaction term: sodium*fever;

title2 'Logistic model 17 (Step 17 added sodium*fever) (p= 0.0868)';

**proc** **logistic** data= Chisambo.aetiolo_final;

class Hypertension fever;

model malariaornot (event= 'Yes')= Hypertension sodium fever

Haemoglobin sodium*fever;

;

**run**;

*kept: Hypertension (p= 0.3949), fever (p= 0.0084), Sodium (p= 0.0241)

Haemoglobin (p= 0.0465);

*removed: sodium*fever (p= 0.9043);

*step 18: added interaction term: fever*Haemoglobin;

title2 'Logistic model 18 (Step 18 added fever*Haemoglobin) (p= 0.0868)';

**proc** **logistic** data= Chisambo.aetiolo_final;

class Hypertension fever;

model malariaornot (event= 'Yes')= Hypertension sodium fever

Haemoglobin fever*Haemoglobin;

;

**run**;

*kept: Hypertension (p= 0.0075), fever (p= 0.9992), Sodium (p= 0.0188)

Haemoglobin (p= 0.0754);

*removed: fever*Haemoglobin (p= 0.4957);

*step 20: Final model- Main effects only;

title2 'Logistic model 20 (Step 20 Final Model-Maim effects only) (p= 0.0868)';

**proc** **logistic** data= Chisambo.aetiolo_final;

class Hypertension (ref= "No") fever (ref= "No");

model malariaornot (event= 'Yes')= Hypertension sodium fever

Haemoglobin;

roc "wasdialysisdone";

roccontrast;

**run**;

;

*kept: Hypertension (p= 0.0075), fever (p= 0.0260), Sodium (p= 0.0116)

Haemoglobin (p= 0.0468);

**PROC** **LOGISTIC** DATA=Chisambo.aetiolo_final OUTEST=betas COVOUT;

CLASS oliguriapresent(ref= 'No') Fever (ref= 'No') Hypertension (ref= 'No')

Season OUTCOMEGOODBAD

Odema1(ref= 'No') lusakaprovincepatient(ref= 'No') peakkidgo2012stage/ PARAM=GLM;

MODEL malariaornot (event= 'Yes') =

fever

Hypertension

Haemoglobin

sodium

peakcreatinine

DurationPD

lengthhospitstay /

lackfit rsquare;

roc "malariaornot";

roccontrast;

**run**;

* Close the PDF file;

ODS PDF CLOSE;

**data** table_3;

length aetiology $**30**;

input aetiology $ count;

datalines;

Malaria 77

Glomerulonephritis 11

Haemolytic_Uremic_Syndrome 8

Posterior_Urethral_valves 6

Sepsis 5

Hypovolaemia 3

Systemic_Lupus_erythematosus 2

Reno_vascular_hypertension 2

Nephrolithiasis 2

Traumatic_injury 2

Herbal_intoxication 1

Malignancy 2

Ureteric_stenosis 1

Brake_fluid_poisoning 1

Enalapril 1

Intravascular_haemolysis 1

Unknown 1

;

**run**;

ODS RTF FILE = 'F:\Working_Folder\Students\2021_23\DrChisambo\Marine.pdf' STARTPAGE = NO;

ODS NOPROCTITLE;

**proc** **freq** data= Table_3;

weight count;

tables aetiology / binomial (level= 'Malaria') ;

**run**;

**proc** **freq** data= Table_3;

weight count;

tables aetiology / binomial (level= 'Glomerulonephritis') ;

**run**;

**proc** **freq** data= Table_3;

weight count;

tables aetiology / binomial (level= 'Haemolytic_Uremic_Syndrome') ;

**run**;

**proc** **freq** data= Table_3;

weight count;

tables aetiology / binomial (level= 'Posterior_Urethral_valves') ;

**run**;

**proc** **freq** data= Table_3;

weight count;

tables aetiology / binomial (level= 'Sepsis') ;

**run**;

**proc** **freq** data= Table_3;

weight count;

tables aetiology / binomial (level= 'Hypovolaemia') ;

**run**;

**proc** **freq** data= Table_3;

weight count;

tables aetiology / binomial (level= 'Systemic_Lupus_erythematosus') ;

**run**;

**proc** **freq** data= Table_3;

weight count;

tables aetiology / binomial (level= 'Reno_vascular_hypertension') ;

**run**;

**proc** **freq** data= Table_3;

weight count;

tables aetiology / binomial (level= 'Nephrolithiasis') ;

**run**;

**proc** **freq** data= Table_3;

weight count;

tables aetiology / binomial (level= 'Nephrolithiasis') ;

**run**;

**proc** **freq** data= Table_3;

weight count;

tables aetiology / binomial (level= 'Traumatic_injury') ;

**run**;

**proc** **freq** data= Table_3;

weight count;

tables aetiology / binomial (level= 'Herbal_intoxication') ;

**run**;

**proc** **freq** data= Table_3;

weight count;

tables aetiology / binomial (level= 'Malignancy') ;

**run**;

**proc** **freq** data= Table_3;

weight count;

tables aetiology / binomial (level= 'Ureteric_stenosis') ;

**run**;

**proc** **freq** data= Table_3;

weight count;

tables aetiology / binomial (level= 'Brake_fluid_poisoning') ;

**run**;

**proc** **freq** data= Table_3;

weight count;

tables aetiology / binomial (level= 'Brake_fluid_poisoning') ;

**run**;

**proc** **freq** data= Table_3;

weight count;

tables aetiology / binomial (level= 'Enalapril') ;

**run**;

**proc** **freq** data= Table_3;

weight count;

tables aetiology / binomial (level= 'Intravascular_haemolysis') ;

**run**;

**proc** **freq** data= Table_3;

weight count;

tables aetiology / binomial (level= 'Unknown') ;

**run**;

* Close the PDF file;

ODS PDF CLOSE;

**data** Table6;

set Chisambo.aetiolo_final;

if typeofdialysisdone= **1** then typeofdialysisdone= '';

**run**;

**proc** **freq** data= Table6;

tables typeofdialysisdone/binomial (level= 'Haemodialysis');

**run**;

**proc** **freq** data= Table6;

tables typeofdialysisdone/binomial (level= 'Peritoneal Dialysis');

**run**;

**data** table_6_Peritoneal;

length Peritoneal_Dialysis_Catheter $**30**;

input Peritoneal_Dialysis_Catheter $ count;

datalines;

Catheter_Block 58

Fluid_Overload 51

Hyperkalaemia 21

Blood_products 16

Uraemia 11

Acidosis 10

Anuria 7

MODs 3

;

**run**;

**proc** **freq** data= table_6_Peritoneal;

weight count;

tables Peritoneal_Dialysis_Catheter / binomial (level= 'Catheter_Block') ;

**run**;

**proc** **freq** data= table_6_Peritoneal;

weight count;

tables Peritoneal_Dialysis_Catheter / binomial (level= 'Acidosis') ;

**run**;

**proc** **freq** data= table_6_Peritoneal;

weight count;

tables Peritoneal_Dialysis_Catheter / binomial (level= 'Anuria') ;

**run**;

**proc** **freq** data= table_6_Peritoneal;

weight count;

tables Peritoneal_Dialysis_Catheter / binomial (level= 'Blood_products') ;

**run**;

**proc** **freq** data= table_6_Peritoneal;

weight count;

tables Peritoneal_Dialysis_Catheter / binomial (level= 'MODs');

**run**;

**proc** **freq** data= table_6_Peritoneal;

weight count;

tables Peritoneal_Dialysis_Catheter / binomial (level= 'Hyperkalaemia');

**run**;

**proc** **freq** data= table_6_Peritoneal;

weight count;

tables Peritoneal_Dialysis_Catheter / binomial (level= 'Fluid_Overload');

**run**;

**proc** **freq** data= table_6_Peritoneal;

weight count;

tables Peritoneal_Dialysis_Catheter / binomial (level= 'Uraemia');

**run**;

**proc** **contents** data= Chisambo.peritoneal;

**run**;

**data** Table7;

set Chisambo.peritoneal;

**run**;

**proc** **freq** data= Table7;

Tables Whoinserted*Typecatheter/norow chisq;

exact fisher;

**run**;

**proc** **freq** data= Table7;

Tables Whereinserted Whereinserted*Typecatheter/norow chisq;

exact fisher;

**run**;

**proc** **sort** data= Table7;

by Typecatheter;

**run**;

**proc** **means** data= Table7 n nmiss mean std median q1 q3 mode min max;

var Daysused;

**run**;

**proc** **means** data= Table7 n nmiss mean std median q1 q3 mode min max;

var Daysused;

by Typecatheter;

**run**;

**proc** **npar1way** wilcoxon correct= no data= Table7;

class Typecatheter;

var Daysused;

**run**;

**proc** **freq** data= Table7;

Tables complication complication*Typecatheter/norow chisq;

exact fisher;

**run**;

**proc** **freq** data= Table7;

Tables Peritonitis Peritonitis*Typecatheter/norow chisq;

exact fisher;

**run**;

**proc** **freq** data= Table7;

Tables omentalblock omentalblock*Typecatheter/norow chisq;

exact fisher;

**run**;

**proc** **freq** data= Table7;

Tables Leak Leak*Typecatheter/norow chisq;

exact fisher;

**run**;

**proc** **freq** data= Table7;

Tables primarynonfunction primarynonfunction*Typecatheter/norow chisq;

exact fisher;

**run**;

**proc** **freq** data= Table7;

Tables Bleeding Bleeding*Typecatheter/norow chisq;

exact fisher;

**run**;

**proc** **freq** data= Table7;

Tables Sepsis Sepsis*Typecatheter/norow chisq;

exact fisher;

**run**;

**proc** **freq** data= Table7;

Tables woundinfection woundinfection*Typecatheter/norow chisq;

exact fisher;

**run**;

**proc** **freq** data= Table7;

Tables dislodged dislodged*Typecatheter/norow chisq;

exact fisher;

**run**;

**proc** **freq** DATA=Chisambo.aetiolo_final;

Tables malariaornot malariaornot*OUTCOMEBADGOOD1/norow chisq;

exact fisher;

**run**;
